# Integrated analysis of behavioral, epigenetic, and gut microbiome analyses in *App*^*NL-G-F*^, *App*^*NL-F*^, and wild type mice

**DOI:** 10.1038/s41598-021-83851-4

**Published:** 2021-02-25

**Authors:** Payel Kundu, Eileen Ruth S. Torres, Keaton Stagaman, Kristin Kasschau, Mariam Okhovat, Sarah Holden, Samantha Ward, Kimberly A. Nevonen, Brett A. Davis, Takashi Saito, Takaomi C. Saido, Lucia Carbone, Thomas J. Sharpton, Jacob Raber

**Affiliations:** 1 Department of Behavioral Neuroscience, Oregon Health & Science University, Portland, OR 97239 USA; 2 Department of Microbiology, Oregon State University, Corvallis, OR 97331 USA; 3 Department of Medicine, Knight Cardiovascular Institute, Oregon Health & Science University, Portland, OR 97239 USA; 4 Department of Neurocognitive Science, Institute of Brain Science, Nagoya City University Graduate School of Medical Sciences, Nagoya, Aichi 467-8601 Japan; 5 Laboratory for Proteolytic Neuroscience, RIKEN Center for Brain Science, Wako, Saitama 351-0198 Japan; 6 Departments of Molecular and Medical Genetics, Oregon Health & Science University, Portland, OR 97239 USA; 7 Departments of Medical Informatics and Clinical Epidemiology, Portland, OR 97239 USA; 8 Division of Genetics, Oregon National Primate Research Center, Beaverton, OR 97006 USA; 9 Department of Statistics, Oregon State University, Corvallis, OR 97331 USA; 10 Departments of Neurology, Psychiatry, and Radiation Medicine, Division of Neuroscience ONPRC, Oregon Health & Science University, Portland, OR 97239 USA; 11 College of Pharmacy, Oregon State University, Corvallis, OR 97331 USA; 12 Department of Behavioral Neuroscience, L470, Oregon Health & Science University, 3181SW Sam Jackson Park Road, Portland, OR 97239 USA

**Keywords:** Neuroscience, Epigenetics and behaviour

## Abstract

Epigenetic mechanisms occurring in the brain as well as alterations in the gut microbiome composition might contribute to Alzheimer’s disease (AD). Human amyloid precursor protein knock-in (KI) mice contain the Swedish and Iberian mutations (*App*^*NL-F*^) or those two and also the Arctic mutation (*App*^*NL-G-F*^). In this study, we assessed whether behavioral and cognitive performance in 6-month-old *App*^*NL-F*^, *App*^*NL-G-F*^, and C57BL/6J wild-type (WT) mice was associated with the gut microbiome, and whether the genotype modulates this association. The genotype effects observed in behavioral tests were test-dependent. The biodiversity and composition of the gut microbiome linked to various aspects of mouse behavioral and cognitive performance but differences in genotype modulated these relationships. These genotype-dependent associations include members of the Lachnospiraceae and Ruminococcaceae families. In a subset of female mice, we assessed DNA methylation in the hippocampus and investigated whether alterations in hippocampal DNA methylation were associated with the gut microbiome. Among other differentially methylated regions, we identified a 1 Kb region that overlapped ing 3′UTR of the *Tomm40* gene and the promoter region of the *Apoe* gene that and was significantly more methylated in the hippocampus of *App*^*NL-G-F*^ than WT mice. The integrated gut microbiome hippocampal DNA methylation analysis revealed a positive relationship between amplicon sequence variants (ASVs) within the Lachnospiraceae family and methylation at the *Apoe* gene. Hence, these microbes may elicit an impact on AD-relevant behavioral and cognitive performance via epigenetic changes in AD-susceptibility genes in neural tissue or that such changes in the epigenome can elicit alterations in intestinal physiology that affect the growth of these taxa in the gut microbiome.

## Introduction

Little is known about the underlying mechanisms of Alzheimer’s Disease (AD) pathology, how it progresses, and how to effectively treat it. A potential contributor to AD pathology is the gut microbiome as it has been shown that the gut microbiome can communicate with the CNS and affect neurobiology and behavioral phenotypes^1–6^. This communication happens via the gut-brain axis, stress hormones, endocrine, sensory neuronal and neuroimmune pathways, and affects behavioral phenotypes, including stress-related behaviors, anxiety, risk avoidance, and depression^1–6^. Alterations in the gut microbiome might be the earliest event in Parkinson disease (PD) pathogenesis, as gut microbiota have been shown to regulate motor impairments and neuroinflammation in a PD mouse model overexpressing α-synuclein^7^. We have shown that the effects of the neurotoxin 1-methyl-4-phenyl-1,2,3,6-tetrahydropyridine (MPTP) on cognitive performance may be, at least in part, mediated by the gut microbiome^8^. MPTP treatment affected the diversity of the gut microbiome. In addition, there were significant associations between microbiome α-diversity and sensorimotor performance, as well as microbiome composition and fear learning. Recent evidence suggests that the gut microbiome might be important in AD as well. Microbiome perturbations using an antibiotic cocktail was associated with reduced Aβ pathology, astrogliosis, and microglial morphology in male, but not female, mice overexpressing hAPP with the Swedish mutation and presenilin 1, and transplants of fecal microbiota of genotype- and age-matched male mice partially restored Aβ pathology and microglial morphology^9^.

Growing evidence indicates that epigenetic mechanisms (e.g., DNA methylation) contribute to AD as well. Changes in DNA methylation, involving the addition or removal of methyl groups at CpG dinucleotides, can impact gene expression and it is shown that dynamic changes in DNA methylation occur across tissues during aging in humans^10^, nonhuman primates^11^, and mice^12,13^. Age-dependent DNA methylation that occur in the brain are associated with the risk of developing various neurological diseases^14^, including AD^15,16^. Studies investigating human brain tissue confirm that both hypo– and hyper-methylation of DNA are associated with AD^17,18^. Consistently, region-specific changes in DNA methylation were reported in the brains of triple-transgenic mouse model of AD (3xTg-AD)^19^. DNA methylation can affect gene expression although it is now clear that the relationship between epigenetic modification and gene expression is not straightforward. Genes whose promoters are hypomethylated might be programmed to overexpress following a secondary stimulus. Amyloid precursor protein containing familial AD mutations and increased generation of Aβ_42_ may constitute such a trigger^20,21^. Unbiased, genome-wide DNA methylation analyses have been used successfully to identify disease pathways in AD over-expressor models^22^. Moreover, they have shown that the cognitive improvement of 3xTg-AD mice following 6 days of treatment is associated with hippocampal changes in DNA methylation in synapse-related and neurogenesis-associated genes^23^. There might also be an association between the gut microbiome and DNA methylation in brain.

In this study, we used two human amyloid precursor protein (hAPP) knock-in (KI) mouse models generated by Dr Saido^24,25^ to investigate the potential associations between AD pathology, the gut microbiome and brain DNA methylation. One of the models only contained Swedish and Iberian mutations (*App*^*NL-F*^), while the other also included a third Arctic mutation (*App*^*NL-G-F*^). Both models express human APP at physiological levels. The *App*^*NL-G-F*^ and *App*^*NL-F*^ show neuropathology and hippocampus-dependent cognitive impairments at 6 or 18 months, respectively. In this study, we behaviorally and cognitively tested 6-month-old *App*^*NL-F*^, *App*^*NL-G-F*^, and C57BL/6J WT mice. After analyzing the gut microbiome in these mice, we also assessed its possible association with behavioral and cognitive performance and whether the genotype predicts this association. Finally, in a subset of female *App*^*NL-G-F*^, *App*^*NL-F*^, and WT mice, we analyzed DNA methylation in the hippocampus and assessed whether alterations in hippocampal DNA methylation were associated with the gut microbiome.

## Results

### Genotype effects on body weights, home cage activity, nest building, activity levels in the elevated zero maze, and performance in the wire hang test

**Figure 1:**
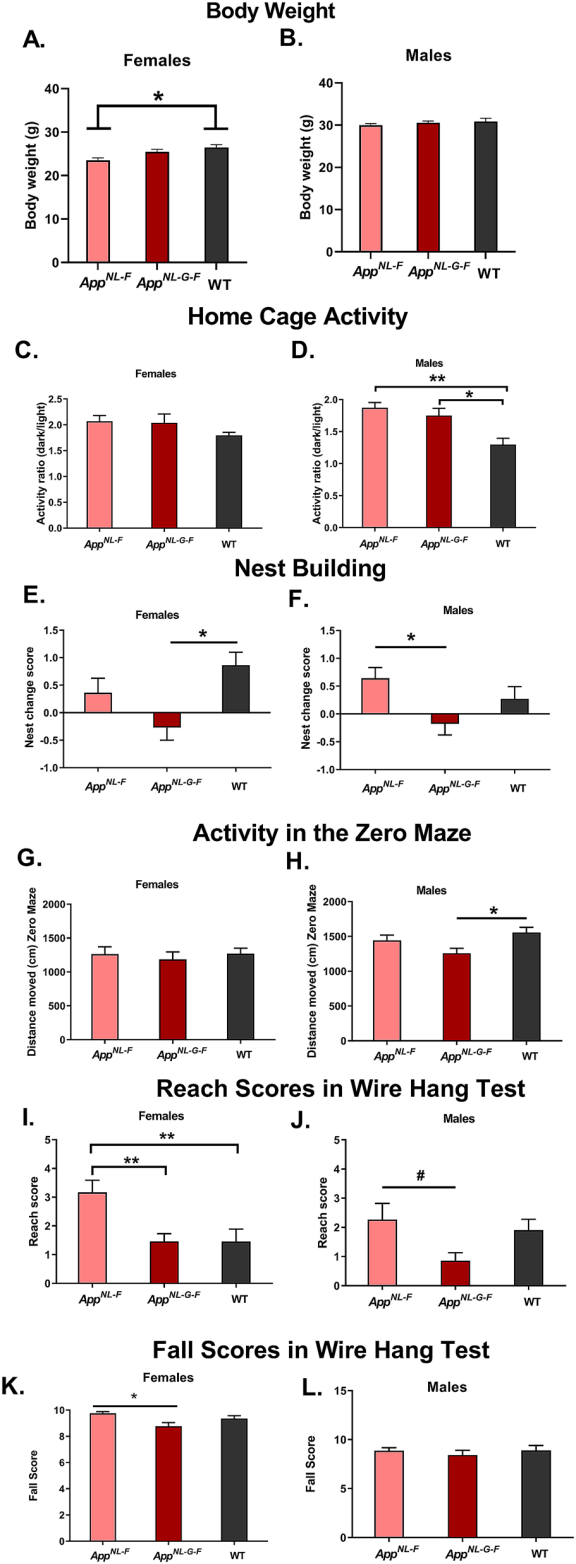
Behavioral performance of *App*^*NL-G-F*^, *App*^*NL-F*^, and WT female (left panels) and male (right panels) mice. The data are shown as mean ± SEM. (**A**) *App*^*NL-F*^ females were lighter than WT females. *p = 0.0039. (**B**) There was no effect of genotype in the male mice. (**C**) There was no effect of genotype on the ratio of circadian activity in female mice. (**D**) *App*^*NL-G-F*^ and *App*^*NL-F*^ males had a higher dark-to-light activity ratio than WT males. *p = 0.0161; **p = 0.0035. (**E**) *App*^*NL-G-F*^ females had a lower nest change score than WT females. *p = 0.0042. (**F**) *App*^*NL-G-F*^ males had a lower nest change score than *App*^*NL-F*^ males. *p = 0.0140. (**G**) There was no effect of genotype on activity of females in the elevated zero maze. (**H**) *App*^*NL-G-F*^ males moved less than WT males in the elevated zero maze. *p = 0.0176. (**I**) *App*^*NL-F*^ females had a higher reach score than *App*^*NL-G-F*^ and WT females in the wire hang test. **p < 0.01. (**J**) *App*^*NL-F*^ males had a higher reach score than *App*^*NL-G-F*^ males in the wire hang test. *p < 0.05. (**K**) *App*^*NL-F*^ females had a higher fall score than *App*^*NL-G-F*^ females in the wire hang test. *p* = 0.0079. (**L**) There was no effect of genotype on fall scores in the males. *App*^*NL-G-F*^ mice: *n* = 13 females and 14 males; *App*^*NL-F*^ mice: *n* = 11 females and 14 males; WT mice: *n* = 11 females and 11 males. C. and D. For home cage activity monitoring, group sizes were: *App*^*NL-G-F*^ mice: *n* = 11 females and 12 males; *App*^*NL-F*^ mice: *n* = 11 females and 10 males; WT mice: *n* = 7 females and 6 males.

The *App*^*NL-G-F*^, but not *App*^*NL-F*^, mice show neuropathology and hippocampus-dependent cognitive impairments at 6 months but whether these effects are sex-dependent and extend to behavioral measures is less clear^24,25^. Therefore, we compared behavioral and cognitive performance in 6-month-old *App*^*NL-G-F*^, *App*^*NL-F*^, and WT mice. There was a significant effect of genotype on body weights in female mice (*F*(2,33) = 5.876, *p* = 0.0066, Fig. 1A), with *App*^*NL-F*^ females being lighter than wild type (WT) females (*p* = 0.0039, Dunnett’s). The body weights of female *App*^*NL-G-F*^ mice were in between those of *App*^*NL-F*^ and WT mice but not significantly different from either of them. In contrast to the female mice, there was no effect of genotype in the male mice (Fig. 1B).

Circadian activity levels were assessed in the home cage. When the ratio of circadian activity, defined as activity during the dark period divided by that in the light period, was analyzed, there was an effect of genotype in the males (*F*(2,25) = 5.398, *p* = 0.0059, Fig. 1D). *App*^*NL-G-F*^ (*p* = 0.0161, Dunnett’s) and *App*^*NL-F*^ males (*p* = 0.0035, Dunnett’s) had a higher dark-to-light activity ratio than WT males. While the same pattern was seen in females, it did not reach significance (Fig. 1C).

The change score in nest building was calculated as the score at time point 1 subtracted from the score at time point 2. Thus, a negative change score indicates decreased nest building over time. A negative change score was seen in *App*^*NL-G-F*^ females (Fig. 1E) and males (Fig. 1F). In females, there was an effect of genotype (*F*(2,32) = 7.382, *p* = 0.0079, Fig. 1E). *App*^*NL-G-F*^ females had a significantly lower change score than WT females (*p* = 0.0042, Sidak’s). There was also an effect of genotype in the males (*F*(2,36) = 4.44, *p* = 0.0189, Fig. 1F). *App*^*NL-G-F*^ males had a significantly lower change score than *App*^*NL-F*^ males (*p* = 0.0140, Fig. 1F).

For total distance travelled in the elevated zero maze, there was no effect of genotype in the females (Fig. 1G). However, there was an effect of genotype on activity levels in the males (*F*(2,37) = 3.926, *p* = 0.0284, Fig. 1H). *App*^*NL-G-F*^ males moved less than WT males (*p* = 0.0176, Dunnett’s). There was no effect of genotype on measures of anxiety, assessed as the percent time spent in the open areas of the maze in females or males (Fig. S1A,B).

For the reaches score, there was an effect of genotype in females (*F*(2,33) = 6.926, *p* = 0.0031, Fig. 1I). *App*^*NL-F*^ females had a higher reach score than *App*^*NL-G-F*^ mice (*p* = 0.0047, Dunnett’s) and WT females (*p* = 0.0065). There was a trend towards an effect of genotype for reaches in the males (*F*(2,37) = 3.009, *p* = 0.0615, Fig. 1J), with a higher reach score in *App*^*NL-F*^ than *App*^*NL-G-F*^ males (*p* = 0.0415, Dunnett’s). For falls, there was an effect of genotype in the females (*F*(2,33) = 5.285, *p* = 0.0102, Fig. 1K). The fall score was higher in *App*^*NL-F*^ than *App*^*NL-G-F*^ females (*p* = 0.0079, Tukey’s). In contrast, there was no effect of genotype of fall scores in the males (Fig. 1L). Thus, there are genotype- and sex-dependent effects on behavioral measures in the *App*^*NL-F*^ and *App*^*NL-G-F*^ mice. While genotype effects were revealed in both sexes in nest building and reach scores in the wire hang test, genotype effects were only revealed in female mice for body weights and fall scores in the wire hang test and only revealed in male mice in home cage activity and activity levels in the elevated zero maze.

### Genotype affected cognitive performance in the Y maze test, but not the object recognition or contextual and cued fear conditioning tests

**Figure 2:**
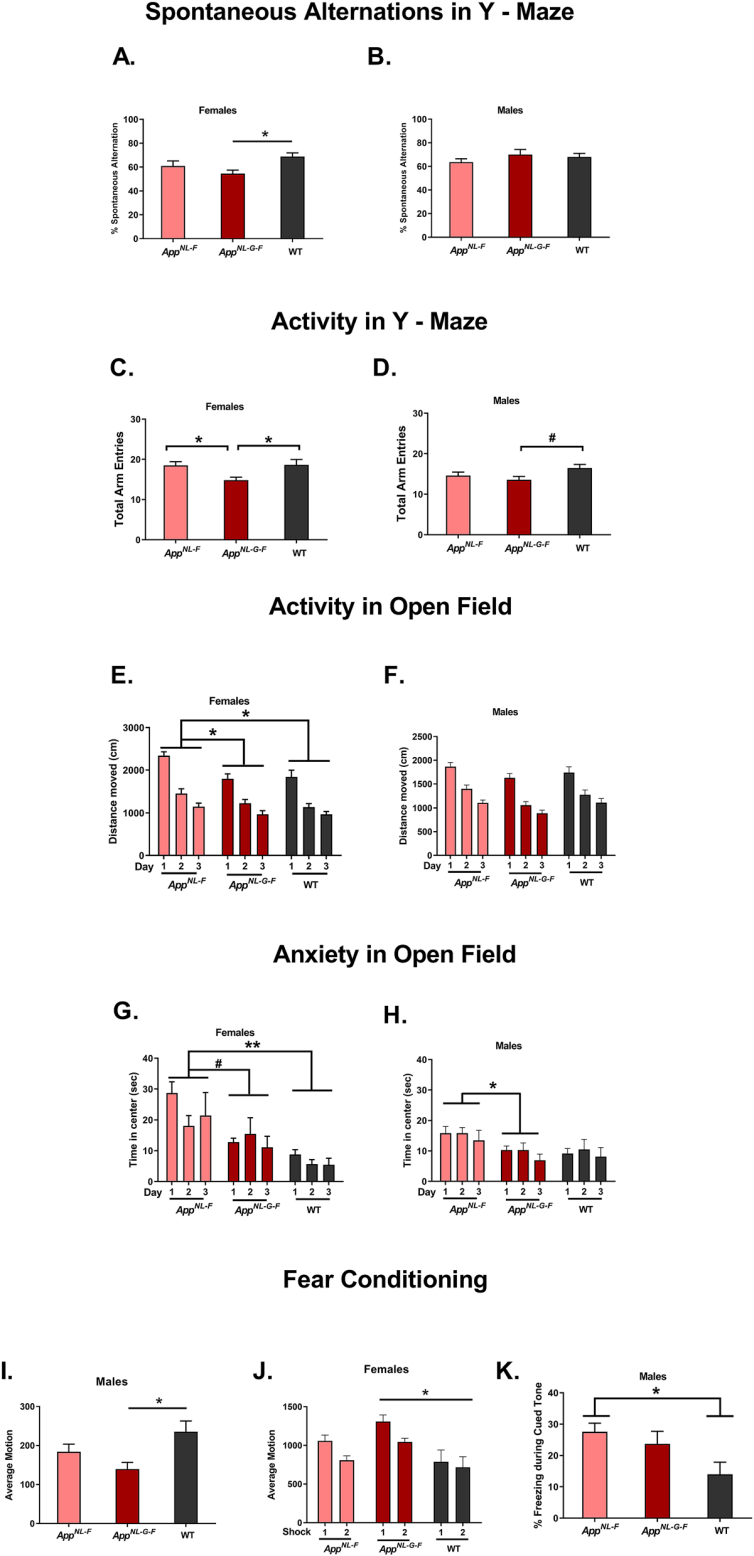
Behavioral and cognitive performance of *App*^*NL-G-F*^, *App*^*NL-F*^, and WT female (left panels) and male (right panels) mice. The data are shown as mean ± SEM. (**A**) The percent spontaneous alternation in the Y maze was lower in *App*^*NL-G-F*^ than WT females. *p = 0.0166. (**B**) There was no effect of genotype on spontaneous alternations in the males. (**C**) The number of entries in *App*^*NL-G-F*^ female mice was lower than in WT and *App*^*NL-F*^ females. *p < 0.05. (**D**) There was a trend towards a lower number of entries in *App*^*NL-G-F*^ than WT males ^#^*p* = 0.0537. (**E**) WT and *App*^*NL-G-F*^ females moved significantly less than *App*^*NL-F*^ females. *p < 0.05. (**F**) There was no effect of genotype in activity levels in the Y maze. (**G**) *App*^*NL-F*^ females spent more time in the center of the open field than WT females. There was a trend towards *App*^*NL-G-F*^ spending more time in the center of the open field than WT females. **p = 0.003; ^#^*p* = 0.063. (**H**) *App*^*NL-G-F*^ males spent less time in the center than *App*^*NL-F*^ males. *p = 0.0364. (**I**) *App*^*NL-G-F*^ male mice moved less than WT male mice during the baseline period in the fear conditioning test. *p = 0.01. (**J**) In females, *App*^*NL-G-F*^ mice moved more during the shocks than WT mice. *p = 0.005. Freezing during the inter-stimulus intervals (ISI’s) was assessed as a measure of fear learning. (**K**) Male *App*^*NL-F*^ mice froze significantly more during the tone in the cued fear memory test than WT male mice. *p = 0.031. *App*^*NL-G-F*^ mice: *n* = 13 females and 14 males; *App*^*NL-F*^ mice: *n* = 11 females and 14 males; WT mice: *n* = 11 females and 11 males.

There was a significant effect of genotype on the percent spontaneous alternations in the females (*F*(2,33) = 4.279, *p* = 0.0223, Fig. 2A). The percent spontaneous alternation was lower in *App*^*NL-G-F*^ than WT females (*p* = 0.0166, Tukey’s). In contrast, there was no effect of genotype on spontaneous alternations in the males (Fig. 2B). There was an effect of genotype on the number of entries in the Y maze in the females (*F*(2,33) = 4.825, *p* = 0.0145, Fig. 2C). The number of entries in *App*^*NL-G-F*^ female mice was lower than in WT (*p* = 0.030, Tukey’s) and *App*^*NL-F*^ females (*p* = 0.032, Tukey’s). There was a trend towards an effect of genotype on the number of entries in the Y maze in the males (*F*(2,37) = 2.575, *p* = 0.0897, Fig. 2D) with a trend towards a lower number of entries in *App*^*NL-G-F*^ than WT males (*p* = 0.0537, Dunnett’s).

Repeated measures ANOVA of total distance traveled in the open field on days 1 through 3 revealed an effect of genotype in females (*F*(2, 33) = 5.225, *p* = 0.011 Fig. 2E), such that WT (*p* = 0.022, Tukey’s) and *App*^*NL-G-F*^ (*p* = 0.023, Tukey’s) females moved significantly less across all three days than *App*^*NL-F*^ females. No such genotype differences were observed in the males (Fig. 2F).

Repeated measures ANOVA of time spent in the center in open field days 1 through 3 revealed an effect of genotype in females (*F*(2,32) 6.668, *p* = 0.004, Fig. 2G). *App*^*NL-F*^ females spent more time in the center than WT females (*p* = 0.003, Tukey’s), and *App*^*NL-G-F*^ females trended towards a similar pattern (*p* = 0.063, Tukey’s). There was also an effect of genotype on time spent in the center of the open field in males (*F*(2,37) = 4.261 *p* = 0.0216, Fig. 2H). *App*^*NL-G-F*^ males spent less time in the center than *App*^*NL-F*^ males (*p* = 0.0364, Tukey’s). There was no effects of genotype on object recognition in female or male mice (Fig. S1C,D).

During fear learning, there was a significant effect of genotype on baseline activity levels (baseline average motion in males (*F*(2, 37) = 4.760, *p* = 0.014 Fig. 2I). *App*^*NL-G-F*^ male mice moved less than WT male mice (*p* = 0.01, Tukey’s). There were no effects of genotype on baseline motion in the females (Fig. S1E). During the tones of fear conditioning acquisition, there was no effect of genotype on freezing in females or males (Fig. S1F,G). To determine potential genotype differences in response to the shock, movement during the shocks was analyzed as well. While an analysis of movement during the two shocks demonstrated no significant effect of genotype in males, there was an effect of genotype in females (*F*(2, 33) = 5.994, *p* = 0.006 Fig. 2J). In females, *App*^*NL-G-F*^ mice moved more during the shocks than WT mice (*p* = 0.005, Tukey’s). Freezing during the inter-stimulus intervals (ISI’s) was assessed as a measure of fear learning. There was no effect of genotype on freezing during the ISIs in females or males (Fig. S1H,I). There was no effect of genotype on contextual fear memory in females or males (Fig. S1J,K). There was no effect of genotype on cued fear memory in females (Fig. S1L). However, there was an effect of genotype on cued fear memory in males (*F*(2, 37) = 3.599, *p* = 0.037 Fig. 2K). Male *App*^*NL-F*^ mice froze significantly more during the tone in the cued fear memory test than WT male mice (*p* = 0.031, Tukey’s). These data show that the genotype effects on cognitive measures in the *App*^*NL-F*^ and *App*^*NL-G-F*^ mice are also sex-dependent. While genotype effects on hippocampus-dependent spontaneous alternations in Y-maze were only seen in females, genotype effects on hippocampus-independent cued fear memory were only seen in males. Genotype effects on activity levels in the open field and response to the shock in fear learning were also sex-dependent and only seen in females, while genotype effects on baseline activity prior to the first tone-shock pairing were only seen in males.

To determine to what extent the behavioral and cogntive tests measure the same underlying abilities, a PCA was performed. The Horn's Parallel Analysis for component retention yielded three factors with eigenvalues > 1.0, which explained a total of 46.244% of the variance among the measures entered into the model (Table 1). Components 1, 2 and 3 explained 17.985%, 16.085% and 12.173% of the variance respectively. Contextual freezing, cued freezing, center duration in the open field, wire hang fall score and wire hang reach score all loaded onto factor 1, indicating a common underlying ability is being assessed in these tasks.

**Table 1:** Principal components analysis: component loadings for behavioral and cognitive measures.

Measure	Component 1	Component 2	Component 3
Contextual freezing	**0.539**	− 0.172	− 0.433
Cued freezing	**0.514**	− 0.244	− 0.361
Nest change score	0.049	0.408	0.323
Open field center duration	**0.659**	0.145	0.023
Percent time with novel object	− 0.332	− 0.167	0.401
Wire hang fall score	**0.548**	0.152	0.392
Wire hang reach score	**0.652**	0.040	0.019
Y-maze arm entries	0.306	0.486	**0.528**
Y-maze percent spontaneous alternations	− 0.174	0.182	− 0.308
Elevated zero maze percent time in open	0.125	− **0.717**	0.409
Elevated zero maze distance travelled	− 0.110	**0.811**	− 0.245

Loadings higher than 0.500 are in bold.

### Gut microbiome associated with cognitive performance in a genotype-dependent fashion

We wanted to explore if the gut microbiome from the three groups of mice associated with the cognitive performance and if the APP genotype modulated this association. Linear models revealed that, for all four metrics assessed, the biodiversity of the gut microbiome (i.e., alpha-diversity) associated consistently with several cognitive measures (Table 2). The percent time freezing during the first tone of the acquisition phase of fear conditioning showed a significant positive association with alpha-diversity (e.g., observed ASVs: *F* = 5.49, *p* = 0.024). Furthermore, two behavioral scores, hippocampus-dependent contextual fear memory (percent freezing in the environment 24 h following training) and time spent exploring the novel object, significantly linked to alpha-diversity in an *App*-genotype dependent manner. The statistical interaction between time spent exploring the novel object and genotype is especially striking, as the relationship between this score and alpha-diversity is negative for the wild type mice, but positive for both the *App*^*NL-F*^ and *App*^*NL-G-F*^ genotypes (observed ASVS: *F* = 7.86, *p* = 0.001; Fig. S2).

**Table 2:** ASVs that had significant associations with both behavior variables and percent methylation of DMRs.

Taxon	Genus	Family	Order	Class	Phylum	Behavior Vars	DMR Genes
ASV0007	Muribaculaceae_Genus	Muribaculaceae	Bacteroidales	Bacteroidia	Bacteroidetes	EZM-time-in-open OF-time-in-center	Glp2r Slc5a10
ASV0038	Bacteroides	Bacteroidaceae	Bacteroidales	Bacteroidia	Bacteroidetes	EZM-time-in-open	ApoE
ASV0051	Alistipes	Rikenellaceae	Bacteroidales	Bacteroidia	Bacteroidetes	EZM-time-in-open	Glp2r Slc5a10
ASV0178	Lachnoclostridium	Lachnospiraceae	Clostridiales	Clostridia	Firmicutes	EZM-time-in-open	Cerkl

**Figure 3:**
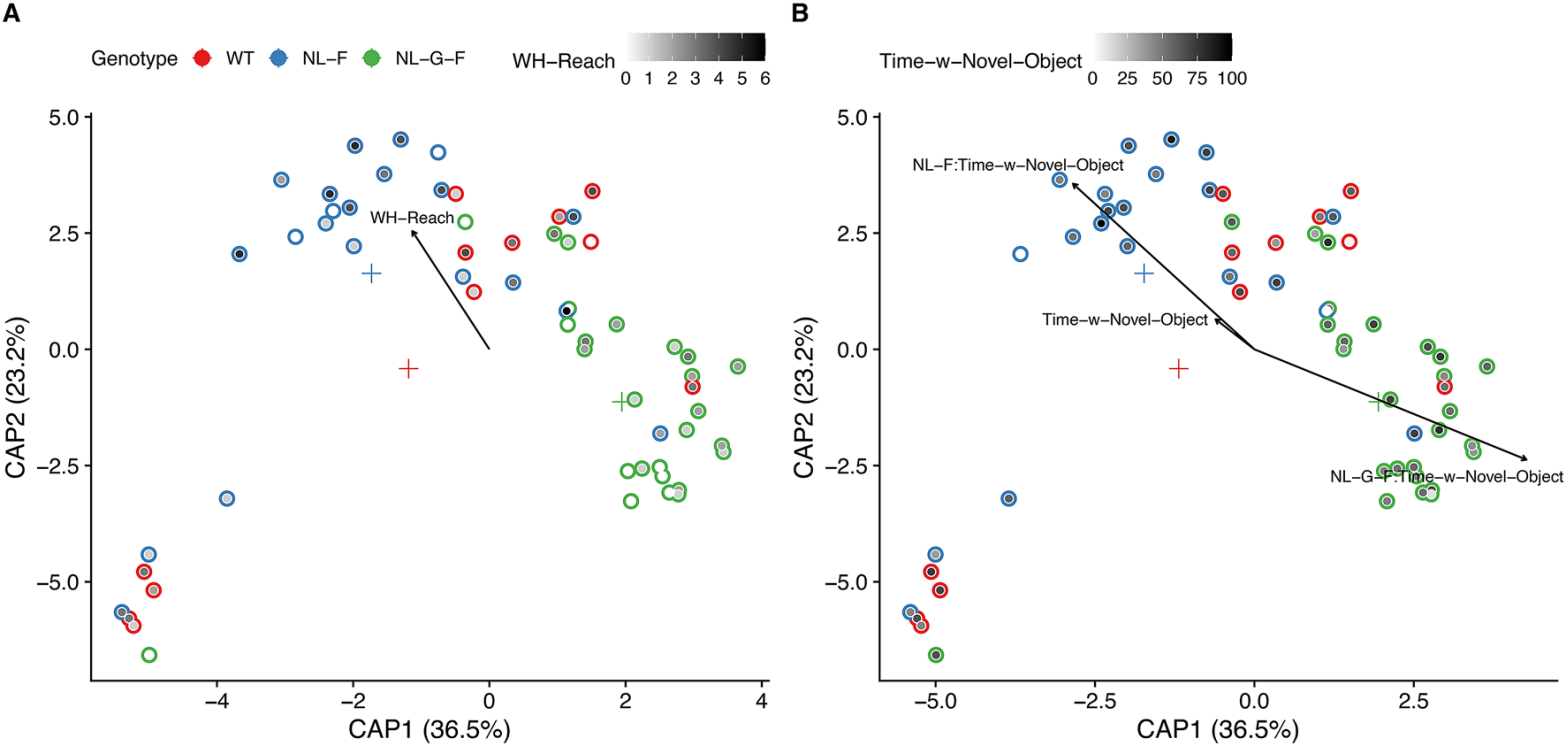
APP genotype (*App*^*NL-G-F*^, *App*^*NL-F*^, WT) modulates the gut microbiome's association, as shown in these dbRDA (‘capscale’) ordinations using the Aitchison distance (Euclidean distance on CLR-transformed abundances). Points represent individual fecal samples. Plus signs mark the centroid for each genotype (colored the same as the point edges). The inside of the points are colored according to percent time freezing during the tone in the wire hang reach score (WH-Reach) or time spent exploring the novel object (Time-w-Novel-Object). PERMANOVA tests support these associations (*p* < 0.05 for both). *App*^*NL-G-F*^ mice: *n* = 13 females and 14 males; *App*^*NL-F*^ mice: *n* = 11 females and 14 males; WT mice: *n* = 11 females and 11 males.

In terms of assessing differences in microbiome composition (*i.e.*, beta-diversity), a PERMANOVA analysis based on the Aitchison distance (Euclidean distance on centered log-ratio transformed counts) revealed that *App* genotype associated with the types of taxa that comprise the gut microbiome (Table 2); *F* = 3.03, *p* = 0.017). The composition of the gut microbiome linked to cognitive performance measures, such as wire hang reach (number of times the animal reached to one of the poles on either end of the wire; *F* = 1.73, *p* = 0.046), independent of genotype (Fig. 3A). Microbiome composition also linked to time spent exploring the novel object (*F* = 1.69, *p* = 0.005) in an *App* genotype-dependent manner (Fig. 3B). Additionally, cage effects do not appear to underlie this association (Suppl. Methods and Suppl. Fig. S4). Collectively, these results indicate that the microbiome correlates with behavior, but that the *App*-genotype modulates this association possibly by altering the types and number of taxa that reside in the gut.

To identify individual microbial taxa that associated with behavioral scores beyond what could already be explained by mouse *App* genotype, we constructed serial compound Poisson generalized linear models that regressed the abundance of all microbiome ASVs against mouse genotype and a specific behavior covariate. After multiple test correction (Bonferroni, *p* = 0.001), these linear models revealed 138 significant taxon-behavior relationships comprised of combinations of 114 different taxa and 10 different behavior scores (Table S5). A little under half 49/114) of the ASVs with significant behavioral associations belong to genera within the Lachnospiraceae family. Ruminococcaceae (26) and Muribaculaceae (13) were the second and third most represented bacterial families in these significant relationships.

### Both App^NL-GF^ and App^NL-F^ mice display drastic changes in hippocampal DNA methylation

**Figure 4:**
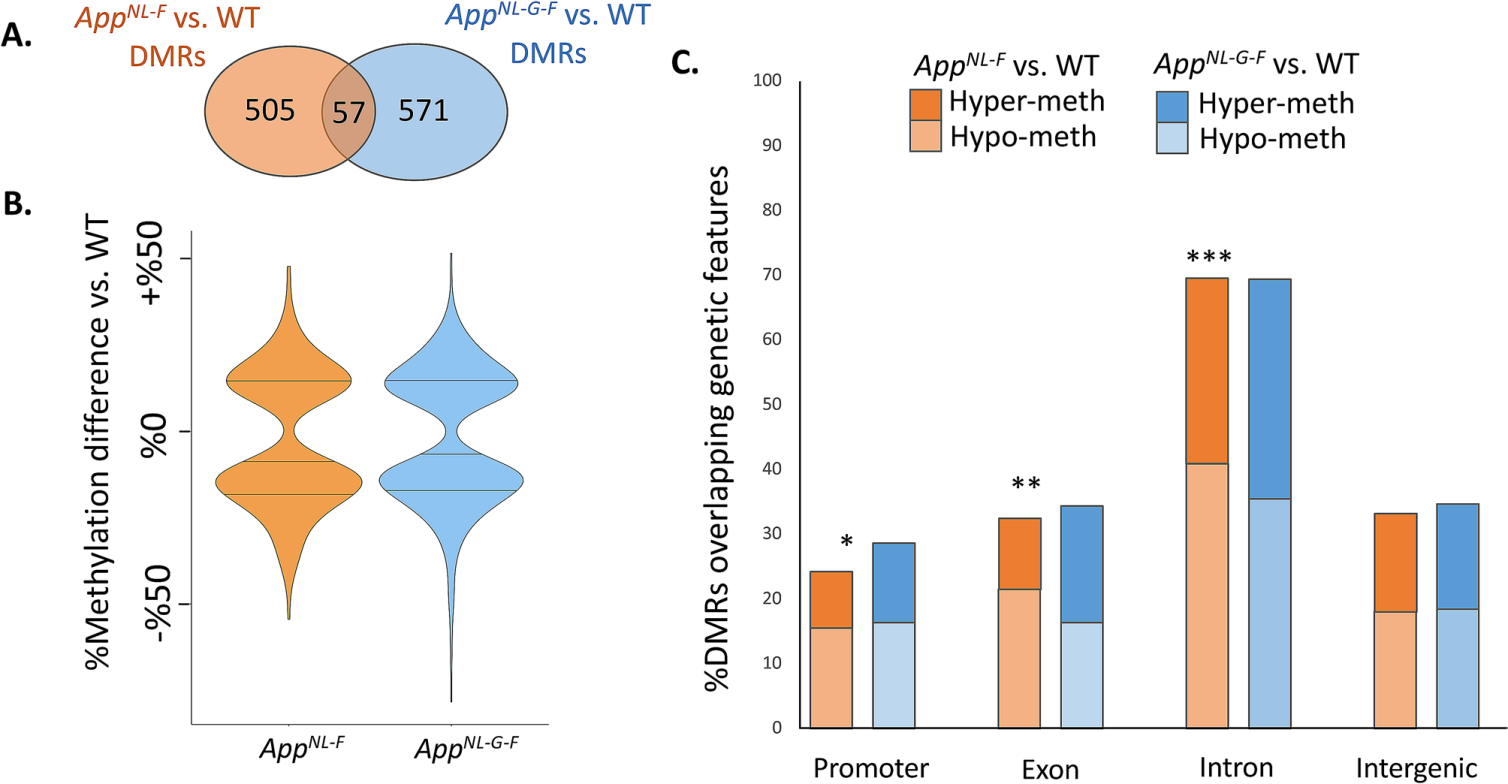
Several differentially methylated regions (DMRs) are found in the genomes of *App*^*NL-F*^ and *App*^*NL-G-F*^ mice when compared to WT. (**A**) A Venn diagram shows the number of unique and shared DMRs in each pair-wise comparison. (**B**) Violin plots demonstrate distribution of methylation differences among the DMRs in each genotype compared to WT controls. (**C**) Percentage of DMRs overlapping each genetic feature is shown. DMRs overlapping more than one feature are included in the counts for each overlapping feature. Proportions of hypo- and hypermethylated DMRs are shown on each bar. Groups with significant deviation from 50:50 split between hypo- and hypermethylated DMRs are marked with asterisk (*p < 0.05, **p < 0.005, and ***p < 0.0005). *App*^*NL-G-F*^ mice: *n* = 5; *App*^*NL-F*^ mice: *n* = 5; and WT mice: *n* = 4.

We used Reduced Representation Bisulfite Sequencing (RRBS) to characterize genome-wide patterns of DNA methylation in the hippocampus of the female *App*^*NL-G-F*^ (*n* = 5), *App*^*NL-F*^ (*n* = 5), and WT control mice (*n* = 5). All samples, except one (WT5), showed low levels of CHG and CHH methylation, where H is A, T or C (CHG: 0.9% ± 0.26 and CHH: 1.1% ± 0.25; mean ± stdev), indicative of successful bisulfite conversion (Table S1). We therefore discarded sample WT5 for all downstream methylation the follow up analyses. We compared genome-wide DNA methylation levels in pair-wise comparisons of each of the *App*^*NL-G-F*^ and *App*^*NL-F*^ mice to age-matched wild-type controls, and identified a total of 628 and 562 unique significant differentially methylated regions (DMRs; *q*-value < 0.05, Table S2), respectively. In total, 67 DMR-containing genes were shared between *App*^*NL-G-F*^ and *App*^*NL-F*^ mice. Of these genes, 57 had fully overlapping DMRs (*i.e.*, methylation change occurring in the exact same region of the gene; Fig. 4A). Surprisingly, the direction (*i.e.*, hyper- or hypomethylation) and amount of methylation change in these shared DMRs were highly similar between *App*^*NL-G-F*^ and *App*^*NL-F*^ mice (*R*^2^ = 0.92, *p* < 2.2e−16; Fig. S4). This similarity in the methylation change at shared DMRs may simply reflect epigenetic differences in *App* KI mouse due to breed differences from WT mice. However, at least some of these DMRs may reflect stable epigenetic changes that emerge before cognitive decline (represented by *App*^*NL-F*^*)* and are maintained after onset of cognitive decline (represented by *App*^*NL-G-F*^*)*.

*App*^*NL-F*^ mice displayed significant bias towards having more hypomethylated DMRs (Fisher’s exact test *p*-value = 0.03), while *App*^*NL-G-F*^ DMRs showed a roughly even split between hypo- and hypermethylated DMRs (Fisher’s exact test *p*-value = 0.241; Fig. 4B). Next, we investigated the proportion of DMRs overlapping different genic structures (promoters, exon or intron) and intergenic regions (> 3 Kb from genes). It should be noted that each DMR may overlap several genetic structures and in such cases, we considered all overlaps in calculating the percentages (Fig. 4C). We found broadly similar patterns of overlap distribution between *App*^*NL-F*^ and *App*^*NL-G-F*^ mice (Fig. 4C). For instance, in both *App*^*NL-F*^ and *App*^*NL-G-F*^ mice, the majority of DMRs (~ 69%) were found overlapping introns while the least common gene structure to overlap DMRs was promoters (< 30%). Hypomethylation was more common than hypermethylation among *App*^*NL-F*^ DMRs that overlapped genic structures (Fisher’s exact test, *p*-value < 0.05), but not in DMRs overlapping intergenic regions (*p*-value = 0.35; Fig. 4C). In *App*^*NL-G-F*^ mice, the ratios of hypo- and hypermethylated DMRs were not significantly deviant from an even split, regardless of the genetic structures they overlapped (Fisher’s exact test, *p*-value > 0.1; Fig. 4C).

### Hippocampal DMRs in App^NL-G-F^ mice are enriched for processes relevant to AD

**Figure 5:**
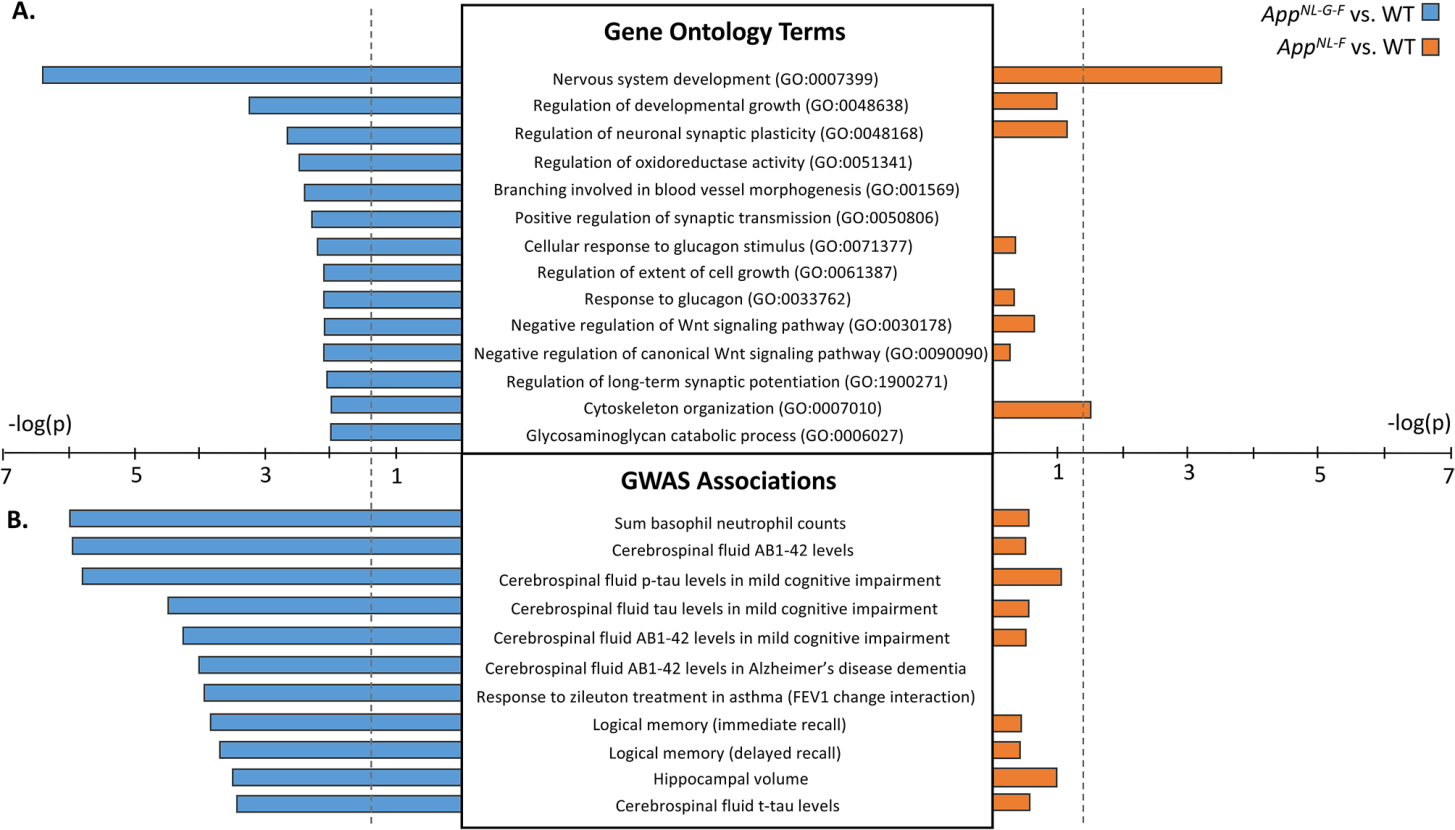
DMR-containing genes in *App*^*NL-G-F*^, but not *App*^*NL-F*^, mice are relevant to human AD. (**A**) The − log_10_(*p*-value) of several AD-related gene ontology terms are showcased from the list of terms with significant enrichment (p < 0.05) in *App*^*NL-G-F*^. (**B**) The –l og_10_(*p*-value) of several AD-related GWAS hits (*q* < 0.05) in *App*^*NL-G-F*^ are shown. *P*-value = 0.05 is marked with dashed vertical line. *App*^*NL-G-F*^ mice: *n* = 5; *App*^*NL-F*^ mice: *n* = 5; and WT mice: *n* = 4.

To investigate the relevance of the DMRs found here to AD in humans, we analyzed enrichment of gene ontology (GO) terms among DMR-containing genes. We found significant enrichment of several GO terms related to AD among the *App*^*NL-G-F*^ DMRs (for example, Regulation of long-term synaptic potentiation (GO:1900271) and Positive regulation of synaptic transmission (GO:0050806); *p*-value < 0.05, Table S3), but not among *App*^*NL-F*^ DMR-containing genes (Fig. 5A, Table S3). Similarly, DMR-containing genes in *App*^*NL-G-F*^, but not *App*^*NL-F*^, mice were significantly enriched for genes associated with several AD-related phenotypes in the National Human Genome Research Institute catalog of published genome-wide association studies (NHGRI-GWAS), for example, Cerebrospinal fluid Aβ1–42 levels in Alzheimer’s disease dementia and Response to zileuton treatment in asthma (FEV1 change interaction) *q*-value < 0.05, Fig. 5B, Table S4).

**Figure 6:**
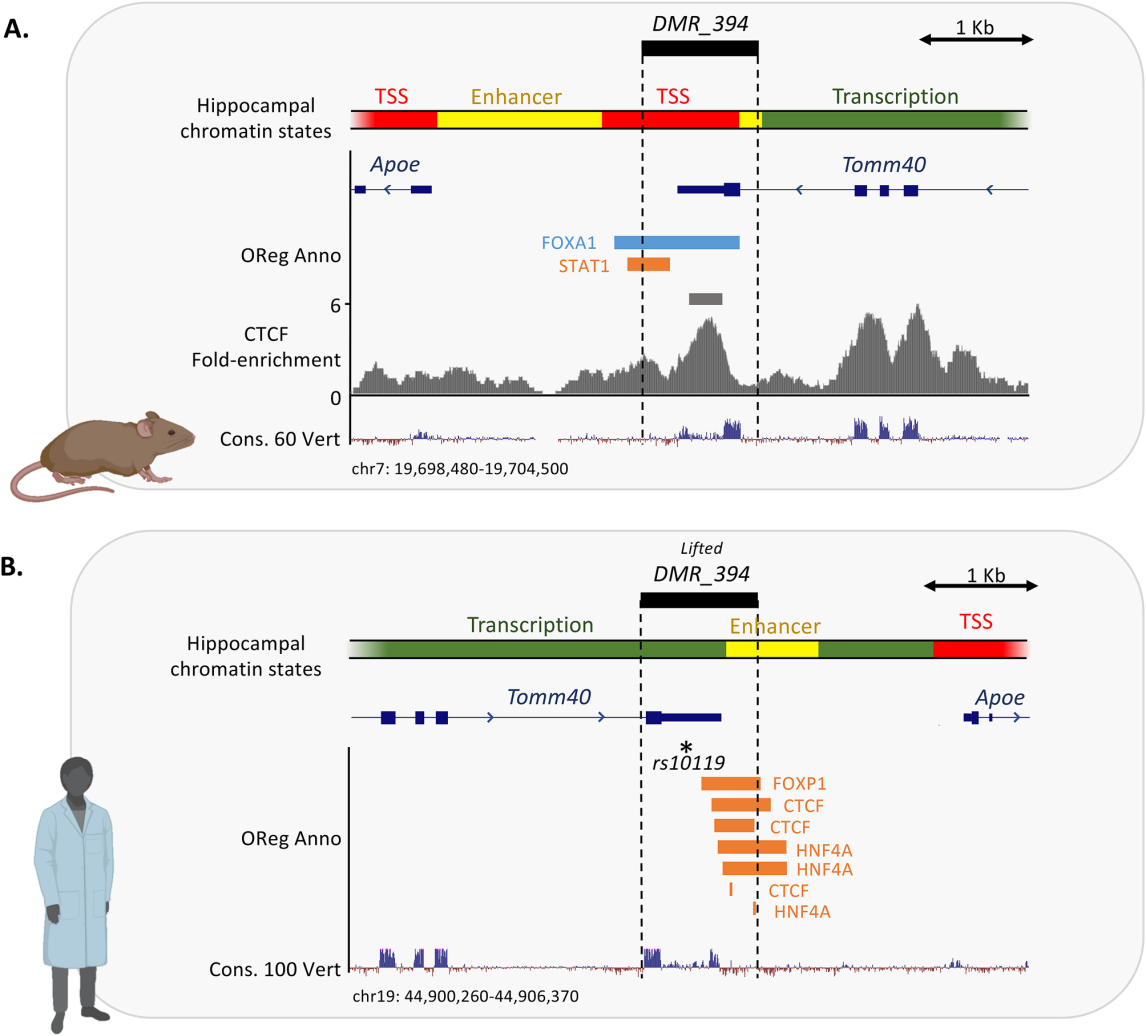
DMR_394 contains a putative hippocampal gene regulatory element conserved in mouse and human. (**A**) The epigenetic landscape at DMR_394 is shown in adult mouse hippocampus tissue. Enhancer states are shown on top. All transcription start site (TSS) states are marked red, all enhancer chromatin states are colored yellow and all transcription-related chromatin states are shaded green. Open Regulatory Annotation (OReg Anno) Database elements are marked blue (regulatory regions) and orange (transcription factor binding site), with the name of the transcription factor believed to bind each element shown next to them. CTCF ChIP-seq fold-enrichment and significant peaks are shown in gray pileup and gray bar, respectively. (**B**) The epigenetic landscape at the human-equivalent of DMR_394 is shown. Chromatin state and OReg Anno colors match those described in panel A.

One of the DMRs that stood out from our analysis because of its potential connection to AD was a 1 Kb region overlapping 3′UTR of the *Tomm40* gene and the promoter region of the *Apoe* gene (Fig. 6A). The *APOE* and *TOMM40* genes are both AD susceptibility genes and interact with each other in modulating AD risk^26^. This *Tomm40-Apoe* DMR (DMR_394) is ~ 21% more methylated in the hippocampus of *App*^*NL-G-F*^ mice, compared to WT controls (*q*-value = 1.04e−21). By overlaying public chromatin state characterizations^27^, we found that DMR_394 displayed epigenetic signatures of transcription start and enhancer activity in hippocampus tissue of adult wild-type mice. Consistently, we found this DMR to overlap known regulatory and transcription factor binding sites from the Open Regulatory Annotation Database^28^. In addition, using public CTCF chromatin immunoprecipitating sequencing (ChIP-seq) data from adult mouse hippocampus^29^ we found evidence that this *Tomm40-Apoe* DMR may be bound by CTCF (Fig. 6A), a protein with crucial functions in regulation of genome organization and gene regulation^30^. All together, these findings indicated that DMR_394 likely contains a regulatory element that is normally active in adult mouse hippocampus. In *App*^*NL-G-F*^ mice, which displayed signs of cognitive decline, this region appears to undergo significant increase in CpG methylation. Given that the affinity of many transcription factors, including CTCF^31^, is negatively impacted by methylation of their binding sites^32^, it is possible that hypermethylation of DMR_394 disrupts binding of regulatory proteins and adversely affects the organization and regulation of genes nearby.

Investigation of human hippocampus chromatin state from publicly available data at the region orthologous to the mouse *Tomm40-Apoe* DMR, revealed signatures of transcription and enhancer activity (Fig. 6B). Furthermore, several CTCF binding sites were reported at this region^28^ suggesting the putative role of this region as a hippocampal regulatory element is conserved in human (Fig. 6B). Of note, this region also includes one of the SNPs (rs10119) significantly associated with AD in the National Human Genome Research Institute catalog of published genome-wide association studies (NHGRI-GWAS^33^), indicating that this regulatory element may be involved in AD in humans (Fig. 6B).

### Integrated gut microbiome hippocampal DNA methylation analysis

**Figure 7:**
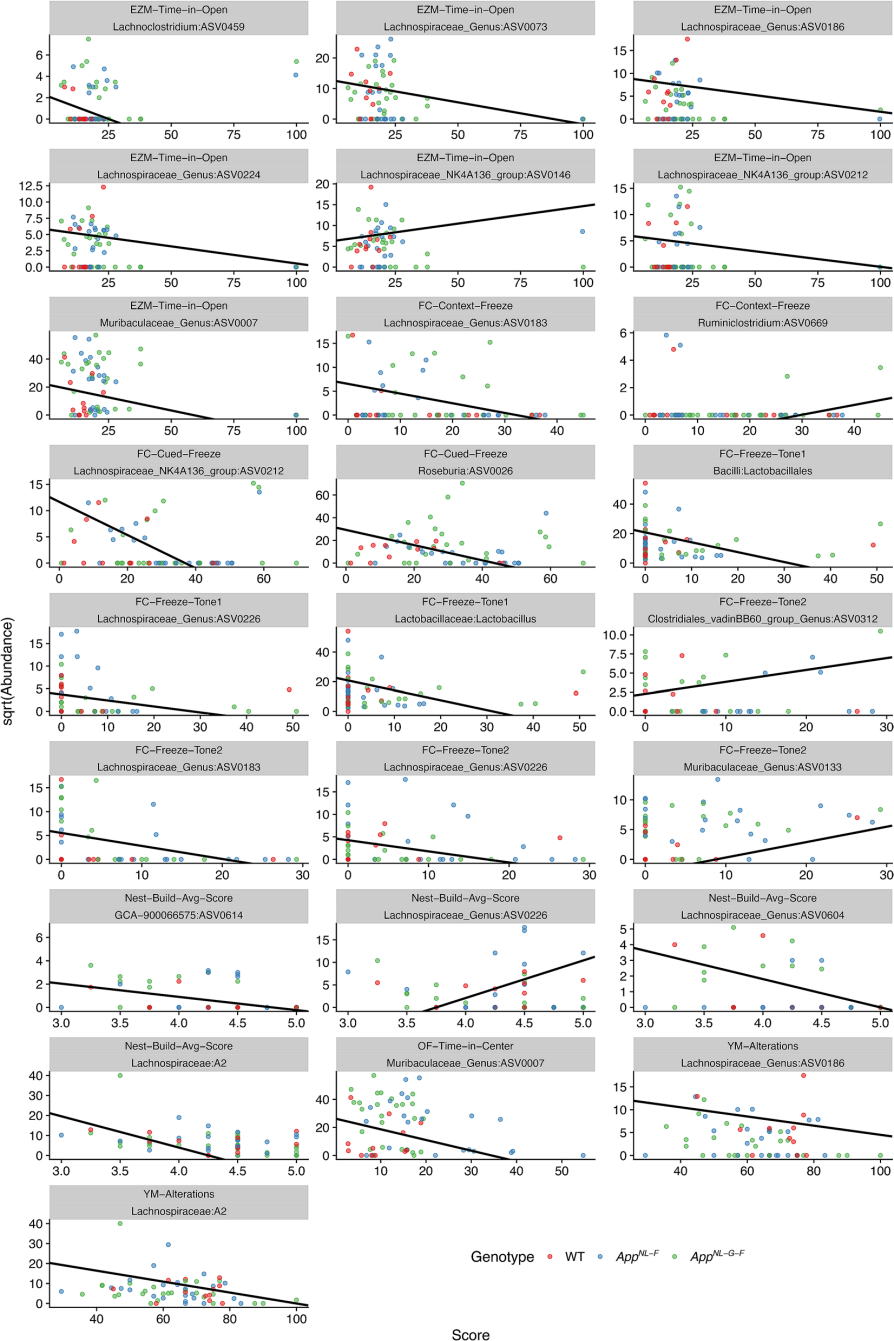
Scatter plots for associations between various behavioral scores (x-axes) and the square root of various taxon abundances (y-axes) found significant utilizing compound Poisson generalized linear models. Black lines indicate the estimated slopes and y-intercepts for each association. Points are colored by mouse genotype: WT (red), *App*^*NL-F*^ (blue), *App*^*NL-G-F*^ (green). *App*^*NL-G-F*^ mice: *n* = 13 females and 14 males; *App*^*NL-F*^ mice: *n* = 11 females and 14 males; WT mice: *n* = 11 females and 11 males.

In order to assess whether there were statistically significant relationships between DMR scores and the microbiome, we conducted analyses analogous to those for assessing the relationships between behavioral scores and the microbiome. Namely, we used PERMANOVA models to test whether DMR scores could predict changes in microbiome composition (beta-diversity) and constructed linear models to test whether taxon abundances could predict DMR scores. To reduce the complexity of this data integration analysis, we prioritized the analysis of 14 DMRs that we selected based on their proximity to AD-associated genes. After stepwise model optimization, none of the 14 DMRs had significant associations with microbiome composition for any beta-diversity metric beyond the variance already explained by mouse genotype. However, after data reduction, model selection, and multiple test correction, linear regression models identified 21 taxa whose CLR-transformed abundance in the gut significantly predicted one or more set of DMR scores. Significant relationships between hippocampal DMRs and individual taxon relative abundances are illustrated in Fig. 7. Of particular note, 4 of these taxa (ASV0007, ASV0038, ASV0051, and ASV0178) also significantly associated with behavioral scores as described above. The observed associations included DMRs linked to apolipoprotein E, which positively associated with ASV0004 (*F* = 47.530, *p* = 0.006) and ASV0137 (*F* = 124.21, *p* = 0.002), which are members of the Muribaculaceae family, as well as apolipoprotein C2, which positively associated with two Lachnospiraceae ASVs (ASV0048; *F* = 12.638, *p* = 0.009; and ASV0056; *F* = 15.759, *p* = 0.005). Additionally, DMRs linked to other AD-associated genes also correlated with specific ASVs. For example, a DMR linked to CERamide Kinase Like (Cerkl) positively associated with a Lachnoclostridium ASV (ASV0178; *F* = 11.269, *p* = 0.012), while an ASV from the Muribaculaceae (ASV0007) negatively associated with a DMR linked to Solute Carrier Family 5 (Sodium/Glucose Cotransporter), Member 10 (Slc5a10) (*F* = 37.966, *p* = 0.004) as well as a DMR linked to Glucagon-Like Peptide-2 Receptor (GLp2r) (*F* = 26.465, *p* = 0.001). Cerkl is a causative gene of retinitis pigmentosa and cone-rod dystrophy retinal disease gene and also associated with microtubule function and found in neurites of neural differentiated cells^34^. Slc5a10 is a glucose transporter gene associated with atherosclerosis risk^35^. GLp2r is especially intriguing as it is expressed in the brain and the gut and involved in spatial cognitive injury following chronic cerebral hypoperfusion^36^. The majority of ASVs that manifest an association with one of these DMRs belong to genera within the Lachnospiraceae family (*n* = 11; tab taxon-methylations selected LMs), indicating that taxa derived from this family of microbes may manifest relatively common associations with hippocampal epigenetic architecture.

## Discussion

In this study, we integrated the results of behavioral, epigenetic, and microbiome analyses in a neurodegenerative disease model in order to investigate the possible impact of these components on AD. The genotype effects in the *App*^*NL-G-F*^, *App*^*NL-F*^, and WT mice on body weights, activity in the home cage, elevated zero maze, and open field, fall scores in the wire hang test, spontaneous alternation in the Y-maze, and cued fear memory were all sex-dependent. Together with the sex differences in pathology seen in mice transgenically expressing App with the Swedish mutation and presenilin 1 with the A246E mutation^37^, these data indicate the sex differences in AD-related genes are not limited to risk factors like APOE but pertinent to dominant AD mutations as well (for review, see^38^).

We observed an effect of genotype on the ratio of circadian activity in the males, with a higher ratio of activity in *App*^*NL-G-F*^ and *App*^*NL-F*^ males than WT males. While a similar trend was seen in females, it did not reach significance. Consistent with an earlier study, we did not observe an overall genotype differences in circadian activity level when only comparing the *App*^*NL-G-F*^ and *App*^*NL-F*^ genotypes^39^.

In the open field, *App*^*NL-F*^ females spent more time in the center than WT females and *App*^*NL-G-F*^ females trended towards a similar pattern. There was also an effect of genotype on time spent in the center of the open field in males, with *App*^*NL-G-F*^ males spending less time in the center than *App*^*NL-F*^ males. We did not see a genotype difference comparing the time *App*^*NL-G-F*^ and WT spent in the more anxiety-provoking center. We did not see a genotype difference in measures of anxiety in the elevated zero maze either. This finding is in contrast to an earlier study that reported decreased anxiety levels in the open field, but increased anxiety levels in the elevated plus maze^40^.

In the Y maze, the percent spontaneous alternation was lower in *App*^*NL-G-F*^ than WT females. In contrast, there was no effect of genotype on spontaneous alternations in the males in our study, similar to the lack if genotype differences reported previously in the Y maze^41^. Our result in female mice is consistent with the cognitive phenotype in the Y maze reported in this genotype^25,42^. However, in these earlier studies in which a cognitive phenotype was observed in the Y maze, male mice were tested by a male researcher in the Y maze and in those studies the Y maze was the first test the mice received. In our study, the Y maze was administered in the third week of testing.

In the open field, WT females moved across less than *App*^*NL-F*^ females. No such genotype differences were observed in the males and when comparing *App*^*NL-G-F*^ and WT males or females. In contrast, lower activity of *App*^*NL-G-F*^ than WT mice were seen in a previous study^41^.

We did not detect an effect of genotype on contextual fear memory, consistent with an earlier study^43^. However, during fear learning, there was a significant effect of genotype on baseline activity levels in males, with male *App*^*NL-G-F*^ mice moving less than WT males. There was also an effect of genotype on movement during the shocks in females, with female *App*^*NL-G-F*^ mice moving more during the shocks than WT mice. These effects were not seen in an earlier study^43^. In our study, there was no effect of genotype on cued fear memory in females but there was an effect of genotype on cued fear memory in males with male *App*^*NL-F*^ mice freezing more during the tone in the cued fear memory test than WT male mice. We did not see a genotype difference in cued fear memory comparing *App*^*NL-G-F*^ and WT female or male mice. In contrast impaired contextual and cued fear memory was reported in *App*^*NL-G-F*^ mice compared to WT mice in an earlier study^44^.

Olfactory cues of male, but not female, researchers is shown to induce stress and related analgesia in rodents^45^. All of our behavioral assays described above were carried out by female researchers and differences in the sex of the researcher and the sex of mice could have contributed to the divergent finding in performance of 6-month-old *App*^*NL-G-F*^ mice in the behavioral tests described above.

Prior work^7,46,47^, indicated that the biodiversity and composition of the gut microbiome are linked to various aspects of mouse cognition and behavior. In this study, we found evidence in support of this notion, but also observed that differences in mouse genotype can modulate these relationships, as evidenced by our beta-diversity analysis that reveal baseline differences in the microbiome based on genotype. For example, the biodiversity of the gut microbiome negatively associated with the amount of time WT mice spent with a novel object, which is an indicator of object recognition memory. However, the biodiversity of *App*^*NL-F*^ and *App*^*NL-G-F*^ genotype mice manifested positive associations with this same measure. Commensurate with this observation, the composition of the gut microbiome manifested striking differences as a function of mouse genotype, with all three genotypes eliciting distinct microbiome compositions from one another. Moreover, the association between the composition of the microbiome and the time a mouse spent exploring a novel object differed in a genotype-dependent manner. Accordingly, APP genotype also affected the relationship between the relative abundance of specific phylotypes in the gut and various behavioral and cognitive measures. These genotype-dependent associations include members of the Lachnospiraceae and Ruminococcaceae families, which our prior mouse model research has also linked to behavior and cognitive performance^8,47,48^. Collectively, these results suggest that the *App*^*NL-F*^ and *App*^*NL-G-F*^ alleles impact the gut microbiome in ways that link to cognitive performance, and support the hypothesis that AD risk alleles may elicit their impact on cognition at least in part by altering the microbiome’s contribution to neurophysiology^49–52^. Future studies are warranted to assess the gut microbiome and its relationship with behavioral and cognitive performance in the same AD model at two distinct time points.

*APOE* and *TOMM40* are AD susceptibility genes^26^. Our analysis revealed 1 Kb a significant Differentially Methylated Region (DMR) overlapping 3′UTR of the *Tomm40* gene and the promoter region of the *Apoe* gene that is ~ 21% more methylated in the hippocampus of *App*^*NL-G-F*^ than WT mice. In addition, using public CTCF chromatin immunoprecipitating sequencing (ChIP-seq) data from adult mouse hippocampus^29^, this *Tomm40-Apoe* DMR was found to be bound by CTCF. CTCF is involved in regulating genome organization and gene regulation^30^. As the affinity of CTCF^31^ is negatively impacted by methylation of its binding site^32^, hypermethylation of this DMR might disrupt binding of regulatory proteins and adversely affects the organization and regulation of genes nearby. Integration of gut microbiome data with hippocampal DNA methylation information at DMRs associated with AD-relevant genes revealed linkages between various ASVs in the gut and a DMR overlapping the *Apoe* gene, which is involved in Reelin signaling. For example, a *Bacteroides*-assigned ASV (ASV0038) negatively associates with this DMR. Various *Bacteroides* species have been associated with health outcomes in both mice and humans. In particular, *B. fragilis* and *B. thetaiotaomicron* have been associated with lower levels of intestinal inflammation as well as various behavioral outcomes such as reduced anxiety, depression, and overall negative behavioral dyregulation^53–55^. Additionally, two ASVs from the Muribaculaea family manifest associations with this same DMR, but in opposing directions. Specifically, ASV0004 negatively associates with this DMR while ASV0137 positively associates with this DMR. While the linkage between Muribaculaceae and neurophysiology has been less well documented relative to other mouse microbiome taxa, prior work has associated the relative abundance of ASVs from this group to various measures of mouse cognition^48,56^. The fact that these relatively closely related ASVs exhibit opposing relationships with this DMR underscores the potential for host-microbe interactions to rapidly diversify and suggests that caution should be employed when leveraging knowledge about close relatives to infer a gut microbe’s biology.

Serine/arginine splicing factors (SRSF) are RNA binding proteins. SRSF11 suppresses tau exon10^57^. Age-dependent loss of SRSF11 in the prefrontal cortex reduces levels of apoE and LRP8 and influences cognitive injury^58^. We also observed associations between gut microbiome and hippocampal methylation nearby more genes implicated in lipoprotein metabolism. For instance, there was a negative relationship between the relative abundance of two Lachnospiraceae ASVs (ASV0048 & ASV0056) and methylation at a DMR nearby apolipoprotein C2, a gene implicated in metabolism of triglyceride-rich lipoproteins (TRLs) by acting as a cofactor of lipoprotein lipase (LPL), the main enzyme that hydrolyses plasma triglycerides on TRL^59^. Members of the Lachnospiraceae are frequently linked to mouse behavior and cognition, though the mechanisms underpinning these associations remain poorly resolved. These associations also exist in human clinical populations—for example, members of the Lachnospiraceae are frequently associated with diagnoses of major depressive disorder across studies^60–63^—suggesting that these taxa may interface with conserved aspects of mammalian neurophysiology. Our novel finding of a linkage between an Apoc2 hippocampal DMR and the relative abundance of these taxa clarifies the potential mechanisms underpinning these longstanding observations. In particular, our observations indicate that these microbes may elicit an impact on AD-relevant behavioral and cognitive performance via epigenetic changes neural tissue AD-susceptibility genes, or that such changes in the epigenome can elicit alterations in intestinal physiology that affect the growth of these taxa in the gut microbiome. Additionally, we uncovered positive relationships between two Lactobacillus ASVs (ASV0040 & ASV0028) and DMRs overlapping the genes Glycogen synthase kinase 3b (GSK3b) and Igfbp4, respectively. GSK3 is a target for AD with promising results of the GSK3 inhibitor SAR502250 on neuroprotection and attenuation of behavioral alterations in AD mouse models^64^.

Members of Lactobacillus have previously been shown to modulate mouse behavioral performance via vagal nerve innervation^65,66^. These data highlight the importance of mouse apoE in human APP KI mouse models. Future efforts are warranted to assess hippocampal DNA methylation in mice expressing both human APP and apoE and lacking the murine APP and apoE counterparts.

By assessing DMRs found in the brains of both KI mice, we found a significant enrichment of several GO terms related to AD among the *App*^*NL-G-F*^ DMRs, but not among *App*^*NL-F*^ DMR-containing genes. In addition, DMR-containing genes in *App*^*NL-GF*^, but not *App*^*NL-F*^, mice were significantly enriched for genes associated with several AD-related phenotypes, including cerebrospinal fluid levels of Aβ_42_ and phosphorylated and total tau, immediate and delayed recall of logical memory, and hippocampal volume. These data are all consistent with the more profound phenotypes of 6-month-old *App*^*NL-G-F*^ than *App*^*NL-F*^ mice and suggest that hippocampal methylation may play a role in regulation of these phenotypes.

The results of this study indicate that the taxonomic diversity and composition of the microbiome correlate with both behavioral/cognitive performance and levels of DNA methylation at certain disease-relevant genes, and that the APP-genotype modulates many of these associations. These data are consistent with the association of microbiome perturbations induced by antibiotics with neuropathological measures in male mice overexpressing hAPP with the Swedish mutation and presenilin 1, and the ability of transplants of fecal microbiota of genotype- and age-matched male mice to partially restore neuropathology^9^. These data suggest that as seen in Parkinson’s disease (PD) and PD models^7^, the gut microbiome might be a contributor to AD in humans and may play an important role in AD models as well. Consistent with this notion, apoE isoform is associated with specific gut microbiome profiles in humans and apoE KI mice^67^. Our results extend these prior observations by revealing connections between behavioral and cognitive performance, epigenetic architecture of the brain, and gut microbiota. However, our associative study is unable to discern cause and effect relationships between connected components, nor does it reveal the functional pathways through which gut microbiota link to the brain epigenome or behavior. Importantly, our efforts have identified individual taxa that are entangled in this tripartite interaction and which thus, serve as strong candidates for future studies to determine whether and how they play causative roles in defining the epigenetic architecture of the brain or the behavioral and cognitive phenotypes observed in APP-genotype mice.

## Methods

### Animals

Human amyloid precursor protein (hAPP) knock-in (KI) mice containing the Swedish and Iberian mutations (*App*^*NL-F*^) or the Arctic mutation included as third mutation (*App*^*NL-G-F*^) generated by Dr Saido^24,25^ on a C57BL/6 J background and age-matched C57BL/6J WT mice were used in the current study. Mice were tested at approximately 6 months of age. Mice were group housed in standard vivarium conditions until the start of the study. They were then singly housed for circadian home cage activity monitoring, as described below, and remained singly housed for the duration of the experiment. The vivarium was maintained at 20–21 °C and food (PicoLab Rodent Diet 20, no. 5053; PMI Nutrition International, St. Louis, MO, USA) was available ad libitum*.* Lights were kept on a 12 h light: 12 h dark cycle. Testing was conducted and data analyzed by an experimenter blind to the genotype of the mice. Procedures complied with the NIH Guide for the Care and Use of Laboratory Animals and with IACUC approval at Oregon Health and Science University (OHSU) and were consistent with the ARRIVE guidelines.

### Behavioral and cognitive assessment group sizes and test order

**Figure 8:**
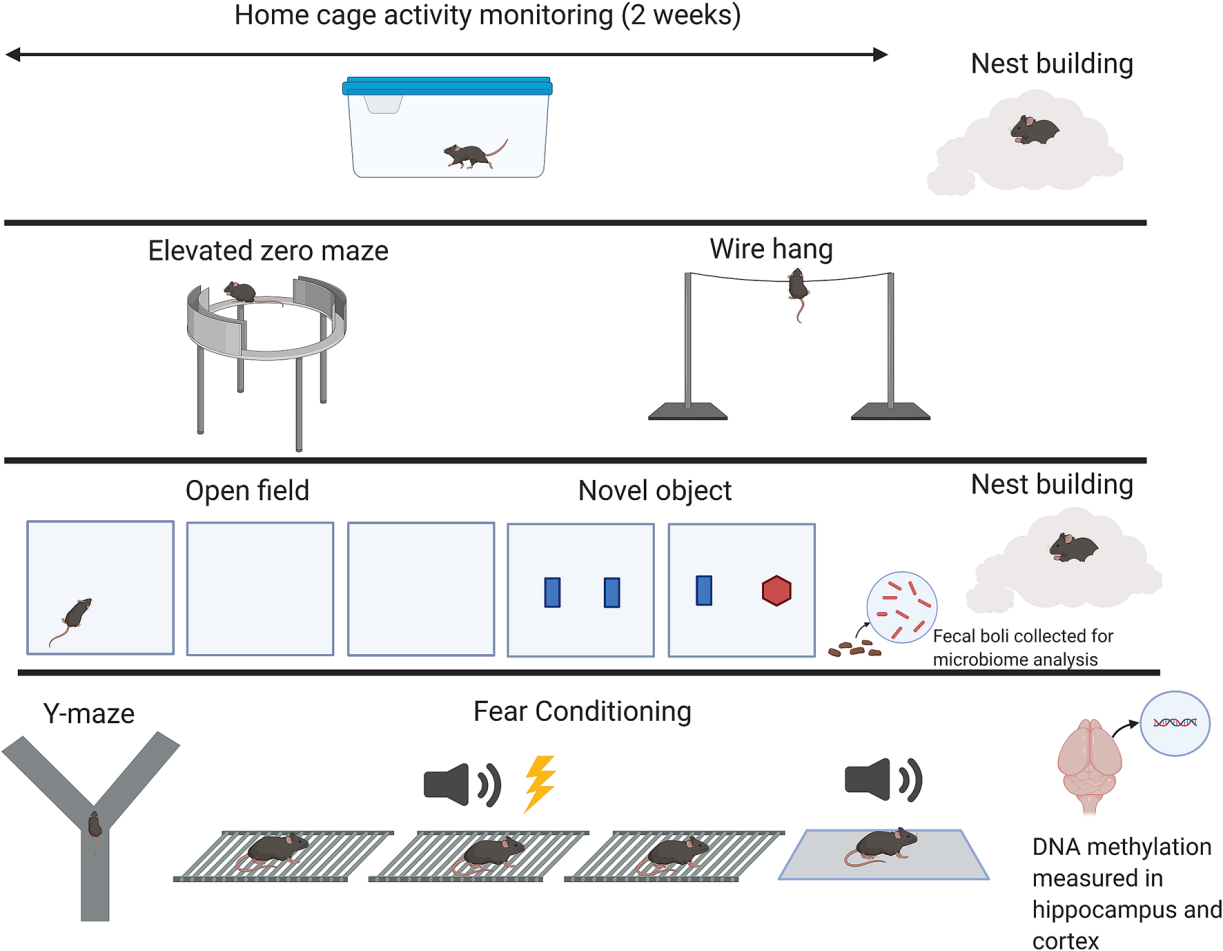
Schematic illustration of the experimental design. Made with Biorender.com.

The group sizes for behavioral and cognitive assessments were as follows: *App*^*NL-G-F*^ mice: *n* = 13 females and 14 males; *App*^*NL-F*^ mice: *n* = 11 females and 14 males; WT mice: *n* = 11 females and 11 males). For home cage activity monitoring, group sizes were smaller due to limitations in the available equipment: *App*^*NL-G-F*^ mice: *n* = 11 females and 12 males; *App*^*NL-F*^ mice: *n* = 11 females and 10 males; WT mice: *n* = 7 females and 6 males. Behavioral and cognitive tests were conducted in the following order as also illustrated in Fig. 8: circadian home cage activity, quality of nest building, measures of anxiety in the elevated zero maze, performance on the wire hang test, measures of activity and anxiety in the open field, object recognition, spontaneous alternation in the Y maze, and contextual fear learning and memory. This order was selected to start with the anticipated least stress-inducing test and end with the anticipated most stress-inducing test. The mazes were surrounded with a white curtain in order to isolate the mice from the surrounding room as well as the experimenter.

### Circadian home cage activity monitoring

Home cage activity was monitored using infrared home-cage activity sensors (Biobserve, St. Augustin, Germany), as previously described^68^. Mice were individually housed during activity monitoring. Activity counts were measured and data were expressed as mean activity count per hour. In addition to analysis of the activity during the light and dark periods, the ratio of circadian activity, defined as activity during the dark period divided by that of the light period was analyzed as well.

### Nest building

Nest building was measured at two time points, once after the conclusion of activity monitoring and again after the conclusion of novel object recognition testing. The protocol described in Deacon, 2006^69^ was used for scoring the quality of the nests, as described^70^. Mice were placed into a clean cage with 3 g of pressed cotton square nestlet. After 24 h, nests were photographed for later scoring on a 5-point nest rating scale. A score of 5 indicates the most complete and well-structured nest, whereas a score of 1 indicates the least functional nest. Scores were assigned in 0.5 point increments.

### Elevated zero maze

Measures of anxiety were assessed in the elevated zero maze as previously described ^71^. The elevated zero maze consisted of two open and two closed areas (each 35.5 cm in length; Hamilton-Kinder, Poway, CA). The closed areas were surrounded by opaque walls (15 cm tall). Mice were placed into the maze into one of the open areas and allowed to explore for a single 10-min trial. Distance moved and the percentage of time spent in the open areas were analyzed with Motor Monitor software (Kinder Scientific, Poway, CA). The zero maze was cleaned with 0.5% acetic acid between trials.

### Wire hang test

A combination of motor function, balance, endurance, and muscle strength was assessed using the wire hang task, adopting the “falls and reaches” method described by van Putten 2011^72^. Mice were placed on a 2 mm metal wire suspended between two metal posts, 35 cm above soft bedding so that they were hanging only by their front paws. The wire was suspended between two vertical metal posts. Initial placement onto the wire was with the forepaws only, though once the trial began, use of back paws was also allowed. Mice started with a “fall score” of 10 and a “reach score” of 0. Over the duration of 180 s, one point was deducted from the fall score each time the animal fell, and one point was gained each time the animal reached to one of the poles on either end of the wire. The time of each fall or reach event was also recorded. At the instance of either event, the timer was paused to place the mouse again on the center of the wire.

### Y-maze

Activity levels and hippocampus-dependent spontaneous alternations were assessed in the Y-maze. The Y-shaped maze (O’ Hara & Co., Ltd, Tokyo, Japan) has raised sides (3.8 cm bottom width, 12.55 cm top width, 12.55 cm height) with plastic, opaque grey arms (37.98 cm length) at a 120° angle from each other. This vendor was selected as the same Y-maze was used in the original study of the *App*^*NL-G-F*^ and *App*^*NL-F*^ mice^25^. Mice were placed in the center of the maze at the beginning of a 5-min trial. The maze was cleaned with 0.5% acetic acid between trials. Performance of the mice was recorded using Ethovision 15 XT software, Noldus Information Technology, Wageningen, The Netherlands. Digital videos were later analyzed using hand scoring to measure the number of arm entries and to calculate the percent spontaneous alternations, a cognitive performance measure based on the innate tendency of rodents to explore a prior unexplored arm the Y-maze. The criteria for an arm entry was when all four limbs were within the arm. The spontaneous alternation percentage was calculated by dividing the number of 3-arm alternations by the number of possible 3-arm alternations and multiplying the value by 100.

### Performance in the open field and object recognition

Exploratory activity and measures of anxiety were assessed in the open field test. The open field consisted of a well-lit square (L 40.6 × W 40.6 × H 40.6 cm) with a central light intensity of 100 lx. Mice were allowed to explore the open field for 5 min during three consecutive days. On day 4, mice were exposed in the open field containing two identical objects for a 15-min trial. The objects were placed 10 cm apart and 15 cm from the adjacent walls of the arena. On day 5, one object was replaced with a novel object and mice were allowed to explore the open field for 15 min. Object exploration was measured manually as time when the mouse’s nose point was within 2 cm of the object. The enclosures were cleaned with 0.5% acetic acid between trials. Performance of mice was tracked using Ethovision 15 XT software. Time spent in the center of the open field was analyzed to assess measures of anxiety. Percent time spent with the novel object was calculated as the number of seconds spent exploring the novel object divided by the total number of seconds spent exploring the novel and familiar objects combined.

### Contextual and cued fear conditioning

Fear conditioning was assessed over the course of 3 consecutive days using a Med Associates mouse fear conditioning system (PMED-VFC-NIR-M, Med Associates, St. Albans, Vermont) and Med Associates VideoFreeze automated scoring system. Mice were placed inside the fear conditioning chamber, where chamber lights were turned on at the beginning of the trial. The first day of testing consisted of a 5-min habituation phase. No tone or shock was administered during habituation. Day two consisted of the acquisition phase, which lasted for a total of 5 min per trial. Following a 120 s baseline habituation period, 2 30-s tones (80 dB) were presented, separated by 60 s inter-stimulus-intervals (ISIs), including after the second tone-shock period. During the last 2 s of each tone, a 0.30 mA foot shock was administered. Twenty-four hours later the hippocampus-dependent contextual fear memory was assessed. Mice were placed into the same chamber as the acquisition phase and remained there for a 5-min trial. The chamber lights were on, but no tones or shocks were presented. Between habituation, acquisition and context trials, the chambers were thoroughly cleaned with 0.5% acetic acid.

Two hours following the contextual fear memory test, hippocampus-independent cued fear memory was assessed. Mice were placed in a novel environment, containing a novel floor texture, angled walls and vanilla extract soaked nestlet fixed to the outside of the wall. Each cued memory trial lasted a total of 6 min. A 180-s baseline period was followed by a 180-s period during which the same tone used during training was presented. No shocks were administered during this phase of testing. Between cued trials, the chambers were thoroughly cleaned with10% isopropanol.

### Gut microbiome analyses

We assessed the microbiome of all animals used for the behavioral and cognitive assessments. Briefly, we used a sterile technique to collect fecal boli samples during the first day of object recognition testing and stored samples at -80 °C until analysis. Frozen fecal samples were shipped on dry ice to Oregon State University, where 16S rRNA gene sequence libraries were prepared using standard procedures^73^ as described^8,74^. Bacterial 16S rDNA sequences were PCR amplified and sequenced as previously described^8,74,75^. Briefly, DNA was extracted from collected fecal pellets using the QIAamp PowerFecal DNA kit (Qiagen, Hilden, Germany) and the V4 region of the 16S rDNA gene was amplified in triplicate using the Earth Microbiome Project 16S PCR protocol. PCR reactions were cleaned with the UltraClean PCR clean-up kit (Qiagen, Hilden, Germany) and samples were diluted to 200 ng of DNA per sample. The prepared libraries were submitted to the Oregon State University Center for Genome Research and Biocomputing (CGRB) for 250 bp paired-end sequencing on an Illumina MiSeq instrument. Quality control, exact sequence variants clustering, and chimera removal were conducted using the dada2 package^76^ for R (R Core Team 2017). Standard approaches were used to quality control 16S sequences and resolve amplicon sequence variants (ASVs) using DADA2^76^.

### Reduced representation bisulfite sequencing (RRBS) library preparation

After the completion of behavioral testing, mice were euthanized via cervical dislocation and brain tissue was dissected. Hippocampi randomly selected from 15 female mice (*App*^*NL-G-F*^ and *App*^*NL-F*^: *n* = 5 mice; WT: *n* = 4 mice) were used to perform DNA methylation analysis. The DNA was extracted and purified using Zymo Genomic DNA Clean & Concentrator-10 kit (Cat.N D410, Irvine, CA). Subsequently, purity of the DNA was verified using Qubit (ThermoFischer, Waltham, MA) and NanoDrop (ThermoFischer, Waltham, MA) analyses. Approximately 80 ng genomic DNA was digested for 2 h with *MspI* restriction enzyme (New England Biolabs, Ipswich, MA), which cuts the DNA at every occurrence of the CCGG sequence, resulting in fragments starting with a CpG site. Libraries were prepared with the NEBNext Ultra II Modules (New England Biolabs) and the NEBNext Methylated Adaptor (New England Biolabs). The ligated DNA was size-selected using Sera-Mag Select magnetic beads (GE Healthcare Life Sciences, Chicago, IL) to produce a final library size of ~ 350 bp. Bisulfite conversion was performed with the EZ DNA Methylation-Gold Kit (Zymo Research, Irvine, CA), followed by PCR amplification with the NEBNext Q5U polymerase and NEBNext Multiplex Oligos for Illumina (New England Biolabs) to barcode each library. The resulting libraries were normalized and multiplexed for single-end (SE X75) sequencing on the Illumina NextSeq 500 platform at the Massively Parallel Sequencing Shared Resource (MSRP) at OHSU.

### RRBS data analysis and identification of differentially methylated regions (DMRs)

The quality of raw reads was assessed with FastQC (v0.11.8^77^), followed by adapter and low-quality base trimming using TrimGalore (v0.6.1^78^) with the “-rrbs” parameter specified. Trimmed reads were aligned to the mouse reference genome from Ensembl (GRCm38 or mm10), using Bismark (v0.20.0^78^) with default parameters. Coverage files output from Bismark were used to obtain CpG methylation information. One WT sample was removed from the analysis as it was a technical outlier with about fivefold higher non-CPG methylation than any other sample, so a total of 14 samples was included in the analysis. The R package methylKit (v1.12.0^79^) was used for differential methylation analysis. CpG sites on unmapped genome assembly contigs were removed, and remaining CpG sites were filtered to exclude CpGs with < 10 × coverage, as well as those in the 99.9th highest percentile of coverages. We used CpG methylation of CpG sites surpassing these coverage thresholds in all samples to perform a PCA analysis in R. We decided to exclude one of the WT samples based on visualization of the PCA analysis, as this sample appeared to be an outlier likely due to incomplete bisulfite conversion.

We used methylKit^79^ to perform pairwise comparisons to identify Differentially Methylated Regions (DMRs) between *App*^*NL-F*^ and WT mice, as well as between *App*^*NL-G-F*^ and wild-type mice. To this end, the genome was tiled into 1 Kb non-overlapping bins, and the CpG methylation rates were averaged within each bin. To calculate p-values, a logistic regression model that utilizes a Chi-square test was used to determine if the model with the treatment vector better predicts the outcome variable (methylation proportion) than the null model. P-values were adjusted for multiple testing (*i.e.*, q-value) via the SLIM method^80^, and overdispersion correction was applied. We only retained DMRs with ≥ 10% methylation difference and *q*-value < 0.05 for downstream analysis.

### Characterization of DMRs in App^NL-F^ and App^NL-G-F^ mice

DMRs were assigned to nearby (≤ 3 Kb) and overlapping genes using GRCm38.92 Ensembl gene annotation along with custom R scripts that utilized the genomation library. We also annotated all gene structures overlapping each DMR (i.e., promoter (3 kb upstream from the transcription start site), exon, intron or intergenic)^81^. When DMRs overlapped more than one genetic feature, all overlaps were considered. We used BEDtools (v2.27.1^82^) to identify DMRs shared between *App*^*NL-F*^ and *App*^*NL-G-F*^ mice. We used the Fisher’s exact test to investigate if the proportion of hyper- and hypo-methylated DMRs deviated significantly from a random 50:50 ratio. Lastly, from each of our pair-wise comparisons (i.e. *App*^*NL-F*^ vs. WT and *App*^*NL-G-F*^ vs. WT), we obtained genes assigned to significant DMRs (*q*-value < 0.05) and used Enrichr^83^ to identify significantly enriched gene ontology terms and to detect over-representation of genes known to contain significant SNPs in human genome-wide association studies (based on the National Human Genome Research Institute catalog of published genome-wide association studies, or NHGRI-GWAS^33^).

### Epigenetic characterization of the Tomm40-Apoe App^NL-G-F^ DMR (DMR_394)

We used chromatin state predictions from adult mouse hippocampus^27^ to characterize the epigenetic landscape at a DMR of interest (DMR_394), which was located at the *Tomm40-Apoe* locus and found to be hypermethylated in *App*^*NL-G-F*^ mice. Briefly, we used the LiftOver tool from the UCSC Genome Browser^84^ to convert the coordinates of chromatin state predictions from the mm9 genome built, to the mm10 built. We then used BEDtools^82^ to identify the chromatin states overlapping DMR_394. In order to identify regulatory regions/transcription factor binding sites (from the Open Regulatory Annotation Database) that overlapped DMR_394, we inspected the corresponding annotation track on the UCSC Genome Browser^84^. To investigate binding of CTCF to the *Tomm40-Apoe* DMR we downloaded raw CTCF ChIP-seq data and aligned them^29^ to the mm10 reference genome using Bowtie2^85^, with default settings. We identified significant CTCF peak using MACS2^84,86^.

We used the LiftOver tool from the UCSC Genome Browser^84^ to identify the region in the human genome (Hg38) corresponding to DMR_394. We inspected overlap of this region with the 15-state chromatin state annotations of the human hippocampus from the Roadmaps Epigenomics Project^87^ as well as regulatory regions/transcription factor binding sites available in the Open Regulatory Annotation Database^28^.

### DNA methylation and behavior correlation analysis

There were 13 samples for which we had behavior and methylation data (*App*^*NL-G-F*^ and *App*^*NL-F*^: *n* = 5 mice; WT: *n* = 3 mice). One additional WT sample (WT_4) was removed from the analysis as it did not have corresponding behavioral data. In order to determine areas of the genome where DNA methylation may correlate with behavior variables, linear models were used with the behavior variable of interest as the dependent variable. The independent variable of each model was the methylation rate in each sample for a particular 1000 bp genomic region. These methylation values were obtained by tiling the genome into 1000 bp non-overlapping regions, with the BEDtools *makewindows* function (v2.27.1^82^), averaging the CpG methylation rates within each tile (excluding CpGs with less than 10 × coverage in a majority of samples), and only including tiled regions which overlapped or lied within 3 Kb of an annotated gene. This resulted in around 100,000 regions being tested.

### Statistical analysis

Behavioral and cognitive data are expressed as mean ± SEM. Performance measures for each behavioral and cognitive test were analyzed using ANOVA or repeated-measures ANOVA. In the case of repeated measures ANOVA, sphericity was tested and Greenhouse–Geisser corrections were used when appropriate. Statistical significance was determined using an error probability level of *p* < 0.05. As we expected sex-dependent genotype effects, the behavioral and cognitive data were analyzed separately in females and males.

A principle components analysis (PCA) was performed for the behavior and cognitive data to determine to what extent these tests measure the same underlying abilities. Performance measures entered into the model were: contextual and cued freezing in the fear conditioning test, the nest change score, the time spent in the center during the first day of the open field test, the percent of time spent exploring the novel object in the novel object recognition test, the fall score and the reach score in the wire hang test, the total arm entries in the Y-maze test, the percent spontaneous alternations in the Y-maze test, the percent time spent in the open areas during the elevated zero maze test, and the distance travelled in the zero maze test. A Horn's Parallel Analysis for component retention was performed and components that were found to have adjusted eigenvalues of greater than 1 were retained in the model. Data were analyzed using SPSS Statistics for Windows (Version 25, Armonk, NY: IBM Corp., Chicago, IL).

For a full account of the microbiome sequence data analysis methods, please see the Supplemental Methods file. The following is a brief summary. All statistics were conducted in *R*^88^. Prior to biodiversity (alpha-diversity) analyses, ASV total abundances in each sample were rarefied (resampled with replacement) to 31,828 counts. In order to assess the relationship between the biodiversity of the microbiome and mouse behavioral test scores, we computed four alpha-diversity metrics for all samples: Observed ASVs, Chao1 index, Shannon index, and Simpson index. The association between these metrics and individual covariates (*e.g.*, behavior test scores, sex) was quantified using linear regression. Prior to microbiome composition (beta-diversity), analyses we applied a centered log-ratio (CLR) transformation on the raw ASV counts in order to account for the fact that microbiome composition is inherently compositional data, and such a transformation has been demonstrated to avoid spurious associations caused by analyzing the raw counts of such data^89^. In order to assess the relationship between the composition of the microbiome and mouse behavioral test scores, we computed the Aitchison distance (Euclidean distance on CLR-transformed counts) for all samples. Permutation analysis of variance (PERMANOVA) statistically quantified the association between compositional distance and mouse behavior. We also assessed the significance of association between the abundance of individual microbial taxa and both mouse behavioral scores and genotype using compound Poisson generalized linear regression^90^. Compound Poisson distributions have been demonstrated to appropriately model the over-dispersion and zero-inflation often observed in microbiome count data. To integrate DMR measures with microbiome data, we developed linear regressions that modeled the variation in DMR for a given marker across individuals as a function of genotype as well as multiple gut taxa that were linked to the marker through a random forest data reduction approach (Supplemental Methods). Model optimization was conducted using stepwise regression based on the Aikake Information Criterion (AIC), wherein models with a full set of covariate terms (ordered by importance score from the random forest model) were reduced to the set of covariates that included the minimal number of terms that could explain the greatest variance^53^. Where we could not build single models for a given hypothesis, we corrected for multiple tests using the Benjamini-Yekutieli correction^91^. For analysis of DNA methylation, DMRs were selected based on *q*-value < 0.05 and methylation difference > 10% and annotated with respect to nearest transcription start site (TSS), promoters, exons, and introns. For the DNA methylation and behavior correlation analysis, following testing, *p*-values were adjusted for multiple testing with the Benjamini–Hochberg method.

## Supplementary Information

Supplementary Methods.

Supplementary Legends.

Supplementary Figure S1.

Supplementary Figure S2.

Supplementary Figure S3.

Supplementary Figure S4.

Supplementary Figure S5.

Supplementary Legends.

Supplementary Table S1.

Supplementary Table S2.

Supplementary Table S3.

Supplementary Table S4.

Supplementary Table S5.
