# Supplementary material for: Integrated analysis of behavioral, epigenetic, and gut microbiome analyses in AppNL-G-F, AppNL-F, and wild type mice
Source: Sci Rep. 2021 Feb 25;11:4678. doi: 10.1038/s41598-021-83851-4 (PMC7907263; doi:10.1038/s41598-021-83851-4)
Supplement: Supplementary file 1 — Supplementary Methods. [file 41598_2021_83851_MOESM1_ESM.pdf]

Integrated analysis of behavioral, epigenetic, and gut microbiome analyses in AppNL-G-F , AppNL-F, and wild type mice

Payel Kundu[1*], Eileen Ruth S. Torres[1*], Keaton Stagaman[2], Kristin Kasschau[2], Mariam Okhovat[3], Sarah Holden[1], Samantha Ward[3], Kimberly A. Nevonen[3], Brett A. Davis[3], Takashi Saito[4], Takaomi C. Saido[5], Lucia Carbone[3], Thomas J. Sharpton[2,9], Jacob Raber1[,10#]

Supplemental Microbiome Methods

Alpha-diversity

Four alpha-diversity metrics were computed for all samples: observed ASVs (a count ASV presence/absence), Chao1 index (an estimation of the actual number of ASVs in a sample given known trends in sampling; [10.1023/A:1026096204727]), Shannon index (a measure of entropy incorporating the number of ASVs and their relative abundances; [10.1002/j.1538-7305.1948.tb01338.x]), and Simpson index (a measure of community evenness, i.e. how equally represented are the ASVs in each sample; [10.1038/163688a0]). After calculating these metrics, the distribution of each metric score was assessed for normality using the Shapiro-Wilk test (*shapiro.test*; R Core Team 2020). Metrics that deviated significantly from a normal distribution (Shannon and Simpson) were transformed using Tukey's Ladder of Powers (*transformTukey*; Salvatore Mangiafico 2020) before incorporation into linear models. To assess the relationship between alpha-diversity and covariates of interest (ten behavioral scores plus mouse genotype), linear models were built using the formula:

$$\text{Alpha-diversity\_metric} \sim \text{Genotype} * (\text{Behavioral\_score1} + \ldots + \text{Behavioral\_score10})$$

These full models were optimized using the function *stepAIC* from the *MASS* package (Venables, W. N. & Ripley, B. D. 2002) to serially add and subtract terms until a minimal Aikake information criterion (AIC) score was reached. Significance of terms in these final models was assessed in a linear regression model.

Beta-diversity

Composition - behavior associations

Prior to microbiome composition (beta-diversity) we applied a centered log-ratio (CLR) transformation on the raw ASV counts as per {doi.org/10.3389/fmicb.2017.02224}. To assess similarity of composition we computed the Aitchison distance (Euclidean distance on CLR-transformed counts) for all samples. We built a distance-base redundancy analysis (dbRDA) model using the function *capscale* from the package *vegan* (Oksanen et al 2019). As with alpha-diversity, the initial models included mouse genotype and ten focal behavioral scores as predictors of the distance matrix (an exact formula as above except replacing the "Alpha-diversity_metric" with the appropriate distance matrix. Again, these full models were optimized by AIC score using the function *ordistep* from the *vegan* package. Furthermore, the ID for the cage that each mouse had been housed in was included as a blocking variable for the *ordistep* selection. Terms in the selected models were assessed for significance using PERMANOVA as implemented by *anova.cca*, also from the *vegan* package (again with cage ID as a blocking variable).

Composition - methylation associations

To assess whether methylation scores from 14 DMRs predicted microbiome composition, we built similar dbRDA models as above. For this analysis we were interested if the methylation data predicted microbiome composition in addition to what mouse genotype already predicted. As such, we build dbRDA models using the following formula:

Distance_matrix ~ DMR_scores1 + … + DMR_scores14 + Condition(Genotype)

Where the "Condition(Genotype)" term tells the model to first account for the variance in microbiome composition explained by mouse genotype and then determine how well the DMR scores explain the remaining variance. As with the behavioral scores, this initial full model was optimized with *ordistep* and the resulting terms in the selected model were assess for significance via PERMANOVA as implemented by *anova.cca*.

Taxon abundances – methylation associations

In order to properly assess whether there were significant associations between particular taxa (not just at the ASV level, but at higher taxonomic levels as well) we first conducted a series of data-reduction steps, and then followed that with model optimization very similar to what we did for the alpha-diversity analyses. To begin our data reduction, we built a random forest model (using the *caret* [Kuhn 2020] and *ranger* [Wright & Ziegler 2017] packages) for each of our 14 sets of DMR scores where the predictor matrix included the CLR-transformed ASV abundances. For each of these random forest models, we extracted the importance score for each taxon, and assessed its significance (*importance_pvalues* from the *ranger* package), using only significant taxa (ordered by importance score) in subsequent models. After this data reduction process, we build 14 full linear models (one for each set of methylation scores) with the general formula:

DMR_scores ~ Genotype + Taxon_abundance1 + … + Taxon_abundanceN

and optimized them with *stepAIC*. Each of these optimized models produces as model-wide p-value, which we subjected to adjustment using *p.adjust* from the base *stats* package with the Benjamini-Yekutieli method. Only terms in models with mode-wide corrected p-values less than or equal to 0.05 were then assessed for significance.

Taxon abundances – behavioral associations

We conducted individual compound Poisson generalized linear regressions (*cpglm* from package *cplm;* Zhang Y 2013) to predict taxon abundances from behavioral test scores. We included mouse genotype as a predictor in each model in order to test if the behavioral score explained addition variance in the taxon abundance beyond what the particular genetic background might. The p-values for the behavioral terms were corrected using the Bonferroni correction and because of the large number of pairwise tests we employed a much stricter correct p-value cutoff of 0.001.

Package references

R Core Team (2020). R: A language and environment for statistical computing. R Foundation for Statistical Computing, Vienna, Austria. URL https://www.R-project.org/.

Salvatore Mangiafico (2020). rcompanion: Functions to Support Extension Education Program Evaluation. R package version 2.3.25.

https://CRAN.R-project.org/package=rcompanion

Venables, W. N. & Ripley, B. D. (2002) Modern Applied Statistics with S. Fourth Edition. Springer, New York. ISBN 0-387-95457-0

Bray, J.R. and Curtis, J.T., 1957. An ordination of upland forest communities of southern Wisconsin. Ecological Monographs (27). Change in Marine Communities: An Approach to Statistical Analysis and Interpretation, pp.325-349.

Sørensen, T.J., 1948. *A method of establishing groups of equal amplitude in plant sociology based on similarity of species content and its application to analyses of the vegetation on Danish commons*. I kommission hos E. Munksgaard.

Jari Oksanen, F. Guillaume Blanchet, Michael Friendly, Roeland Kindt, Pierre Legendre, Dan McGlinn, Peter R. Minchin, R. B. O'Hara, Gavin L. Simpson, Peter Solymos, M. Henry H. Stevens, Eduard Szoecs and Helene Wagner (2019). vegan: Community Ecology Package. R package version 2.5-6. https://CRAN.R-project.org/package=vegan

Max Kuhn (2020). caret: Classification and Regression Training. R package version 6.0-86. https://CRAN.R-project.org/package=caret

Marvin N. Wright, Andreas Ziegler (2017). ranger: A Fast Implementation of Random Forests for High Dimensional Data in C++ and R. Journal of Statistical Software, 77(1), 1-17. doi:10.18637/jss.v077.i01

Zhang Y (2013). “Likelihood-based and Bayesian Methods for Tweedie Compound Poisson Linear Mixed Models.” _Statistics and Computing_, *23*, 743-757.
