# Supplementary material for: Integrated analysis of behavioral, epigenetic, and gut microbiome analyses in AppNL-G-F, AppNL-F, and wild type mice
Source: Sci Rep. 2021 Feb 25;11:4678. doi: 10.1038/s41598-021-83851-4 (PMC7907263; doi:10.1038/s41598-021-83851-4)
Supplement: Supplementary file 2 — Supplementary Legends. [file 41598_2021_83851_MOESM2_ESM.docx]

**Supplementary Figure Legends**

**Fig. S1.** Behavioral and cognitive performance of *App^NL-G-F^*, *App^NL-F^*, and WT female and male mice. **A/B.** There was no effect of genotype on measures of anxiety, assessed as the percent time spent in the open areas of the maze in females or males. **C/D.** There were no effects of genotype on object recognition in female or male mice. **E.** There were no effects of genotype on baseline motion in the females. **F/G.** During the tones of fear conditioning acquisition, there was no effect of genotype on freezing in females or males. **H/I.** There was no effect of genotype on freezing during the ISIs in females or males. **J/K.** There was no effect of genotype on contextual fear memory in females or males. **L.** There was no effect of genotype on cued fear memory in females.

*App^NL-G-F^* mice: *n* = 13 females and 14 males; *App^NL-F^* mice: *n* = 11 females and 14 males; WT mice: *n* = 11 females and 11 males.

**Fig. S2**. Shared DMRs between *App^NL-G-F^* and *App^NL-F^* display similar direction and level of methylation change relative to age-matched WT controls. The %methylation changes (relative to wild-type (WT) controls) is shown for 57 shared DMRs between *App^NL-G-F^* (x-axis) and *App^NL-F^* (y-axis).

*App^NL-G-F^* mice: *n* = 5; *App^NL-F^* mice: *n* = 5; and WT mice: *n* = 4.

**Fig. S3.** The amount of time a mouse spent exploring the novel object significantly associated with the diversity of its gut microbiome, in a genotype-dependent manner. For the wild type mice (red), this association is negative for the *App^NL-F^* (blue) and *App^NL-G-F^* (green) genotypes, this association is positive (*p* < 0.005 for this interaction for all four alpha-diversity metrics).

*App^NL-G-F^* mice: *n* = 13 females and 14 males; *App^NL-F^* mice: *n* = 11 females and 14 males; WT mice: *n* = 11 females and 11 males.

**Fig. S4.** Cohousing does not appear to be a strong driver of microbiome composition, as shown in these dbRDA (‘capscale’) ordinations using the Aitchison distance (Euclidean distance on CLR-transformed abundances). These are the exact same ordinations used in Figure 4, with different metadata represents. Text (panels A & B) and points (panels C & D) represent individual fecal samples. Plus signs mark the centroid for each genotype (colored the same as the text and points; all panels). The text (h1 – h14) are cage IDs (panels A & B). Gray dotted lines connect cage mates (panels B & D). Circular points are female mice and triangular points are male mice (panels C & D).

*App^NL-G-F^* mice: *n* = 13 females and 14 males; *App^NL-F^* mice: *n* = 11 females and 14 males; WT mice: *n* = 11 females and 11 males.

**Fig. S5.** Scatter plots for associations between various taxon abundances (x-axes) and methylation level at differentially methylated regions (DMRs) (y-axes) found significant utilizing AIC-selected linear models. Black lines indicate the estimated slopes and y-intercepts for each association. Points are colored by mouse genotype: wild type (red), *App^NL-F^* (blue), *App^NL-G-F^* (green).

Taxon data: *App^NL-G-F^* mice: *n* = 13 females and 14 males; *App^NL-F^* mice: *n* = 11 females and 14 males; WT mice: *n* = 11 females and 11 males.

Methylation data: *App^NL-G-F^* mice: *n* = 5; *App^NL-F^* mice: *n* = 5; and WT mice: *n* = 4.

**Supplemental Tables**

**Supplemental Table S1.** Statistical results from microbiome data analyses, including biodiversity and compositional associations with genotype and behavior, as well as regression analyses that link taxa to behavior covariates or DMRs.
