# Supplementary figures and images for: Integrated analysis of behavioral, epigenetic, and gut microbiome analyses in AppNL-G-F, AppNL-F, and wild type mice
Source: Sci Rep. 2021 Feb 25;11:4678. doi: 10.1038/s41598-021-83851-4 (PMC7907263; doi:10.1038/s41598-021-83851-4)

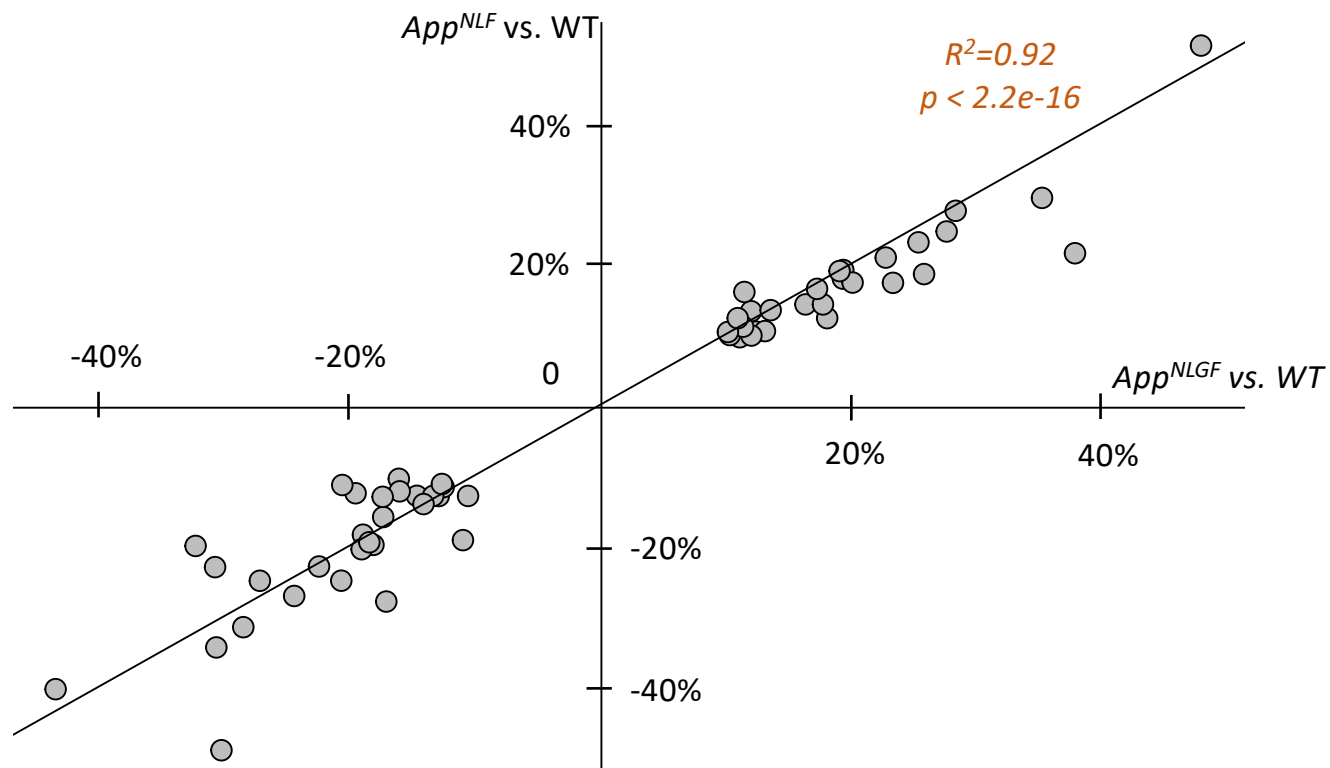

Supplement: Supplementary file 4 — Supplementary Figure S2. [file 41598_2021_83851_MOESM4_ESM.pdf]

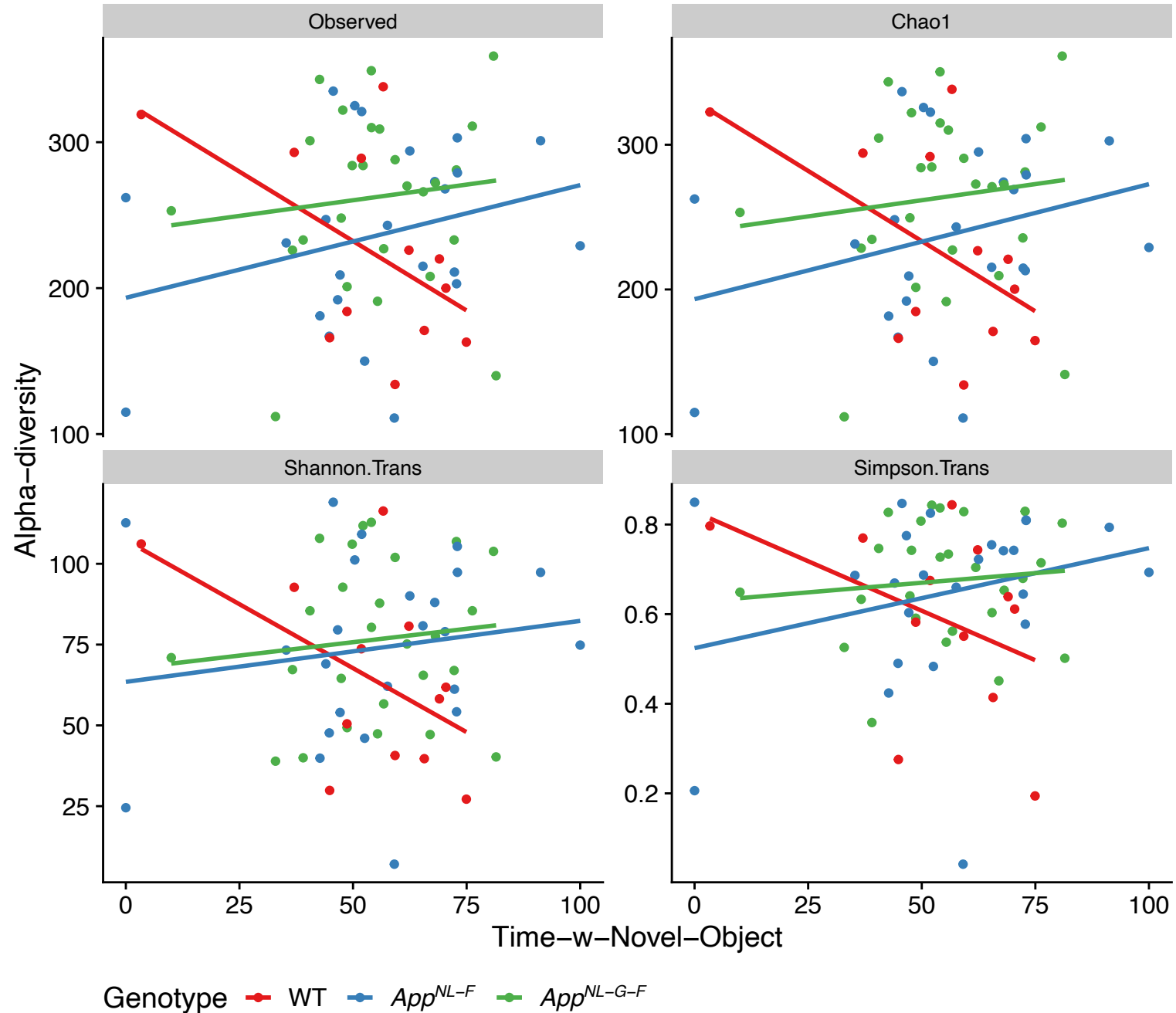

Supplement: Supplementary file 5 — Supplementary Figure S3. [file 41598_2021_83851_MOESM5_ESM.pdf]

**A**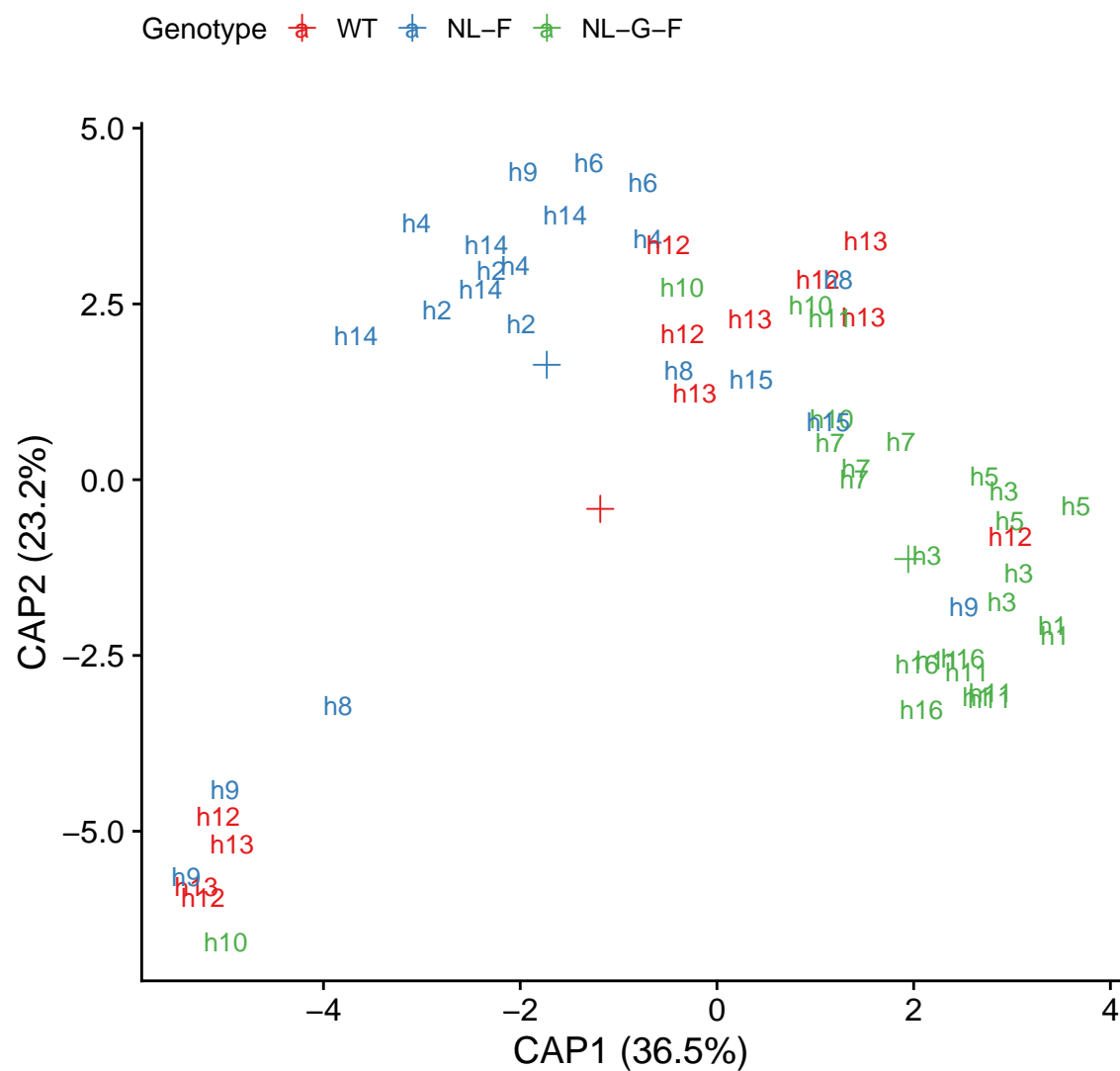**B**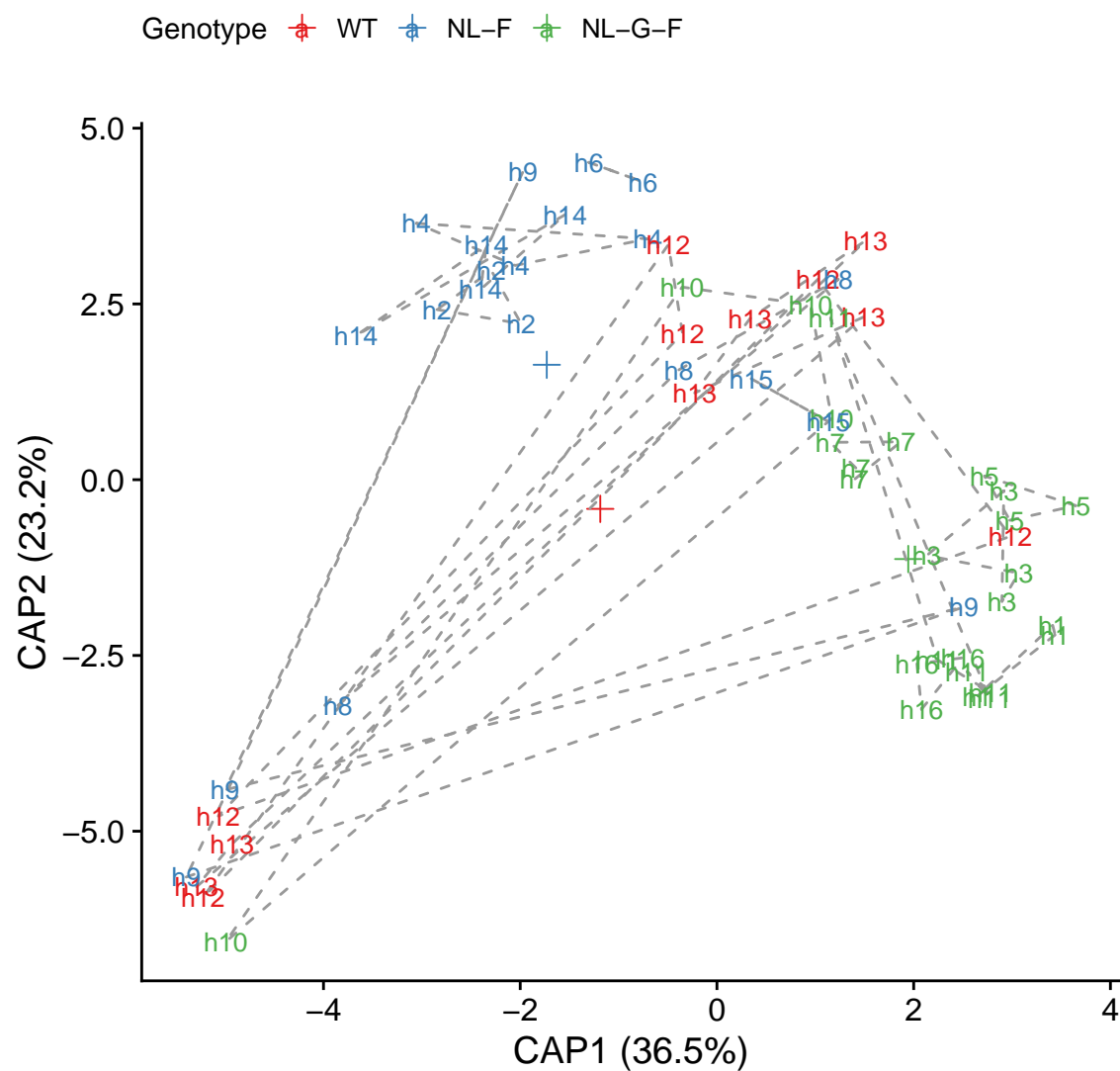**C**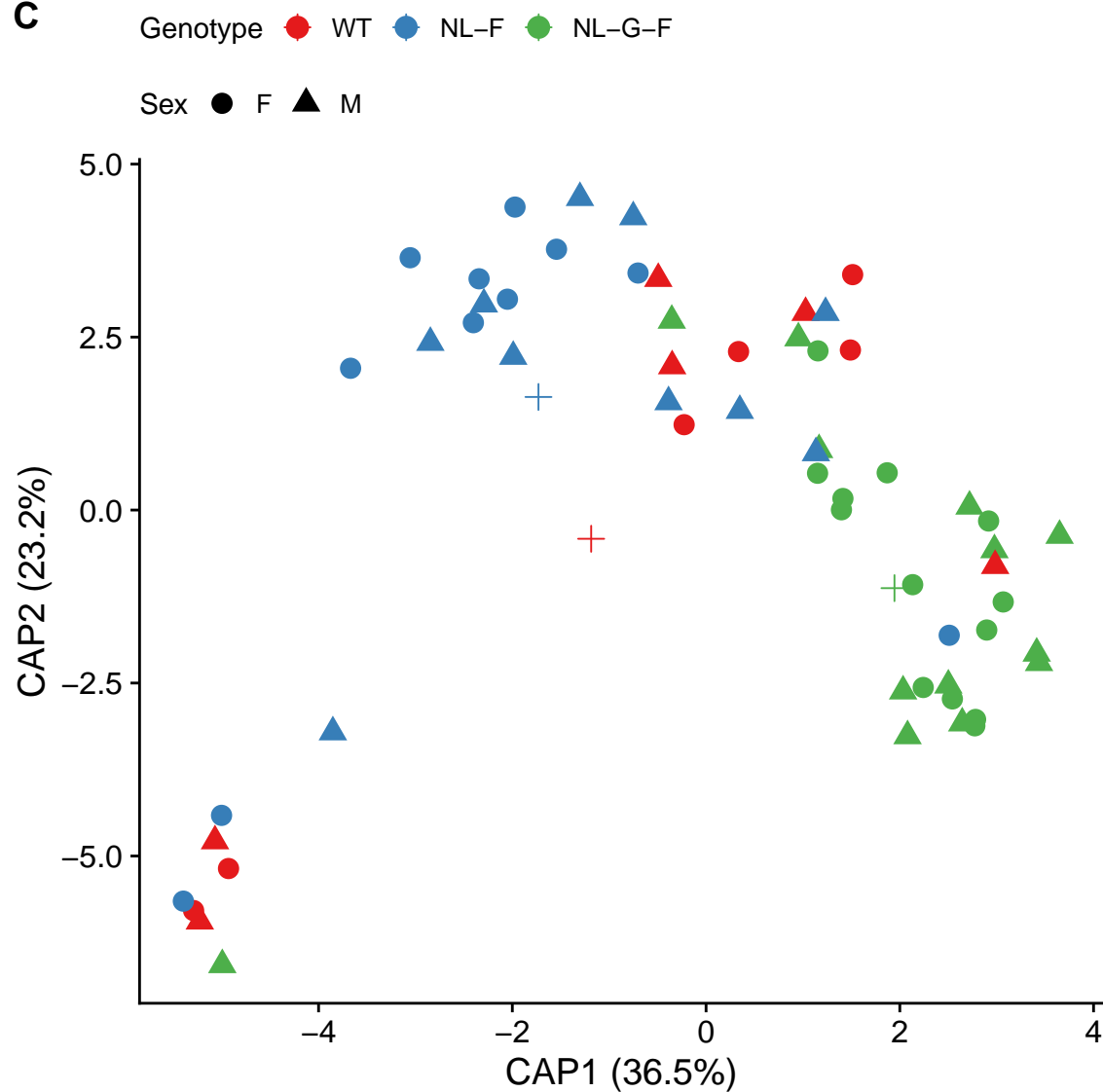**D**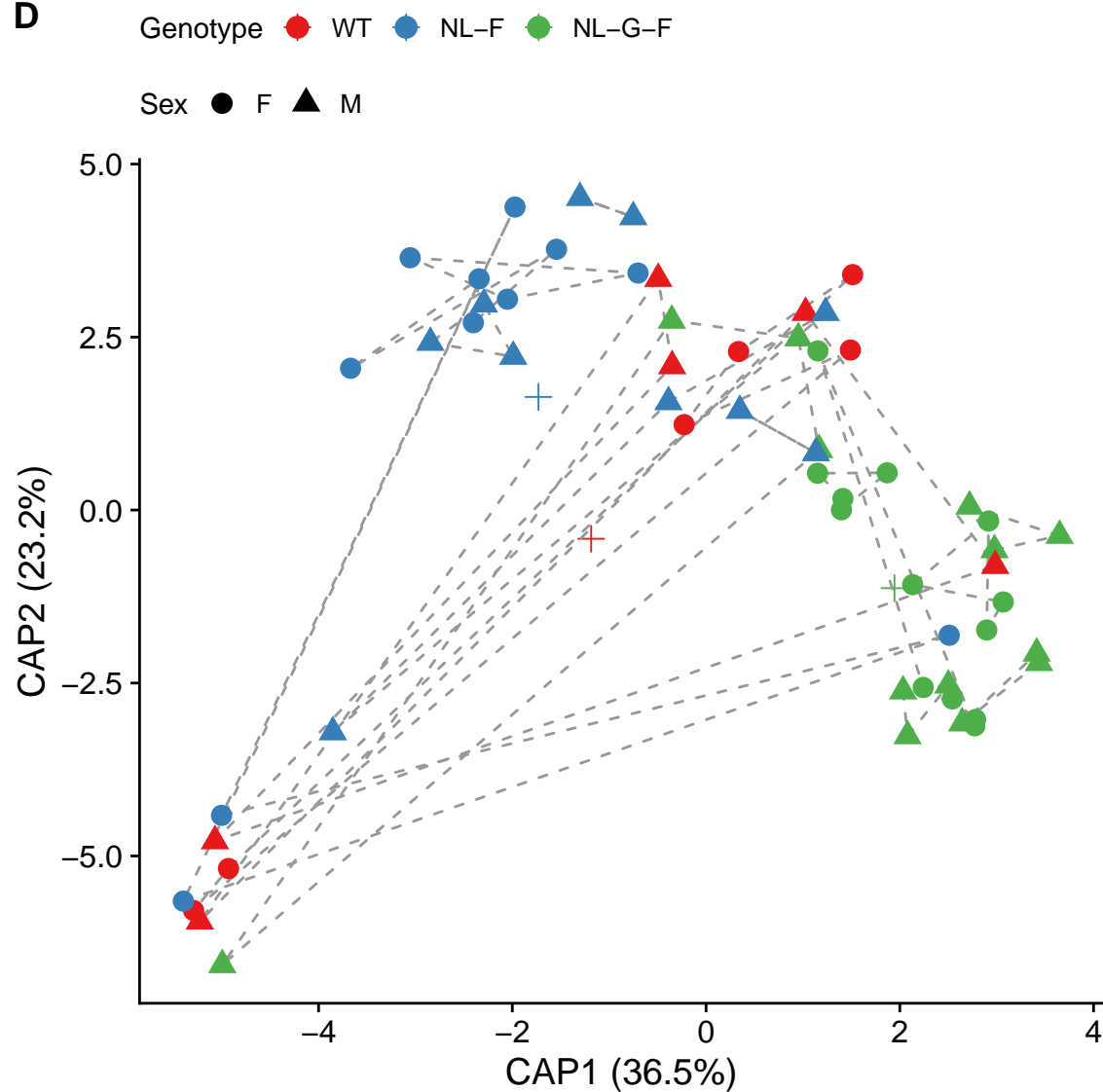

Supplement: Supplementary file 6 — Supplementary Figure S4. [file 41598_2021_83851_MOESM6_ESM.pdf]
