# Supplementary material for: Integrated analysis of behavioral, epigenetic, and gut microbiome analyses in AppNL-G-F, AppNL-F, and wild type mice
Source: Sci Rep. 2021 Feb 25;11:4678. doi: 10.1038/s41598-021-83851-4 (PMC7907263; doi:10.1038/s41598-021-83851-4)
Supplement: Supplementary file 7 — Supplementary Figure S5. [file 41598_2021_83851_MOESM7_ESM.pdf]

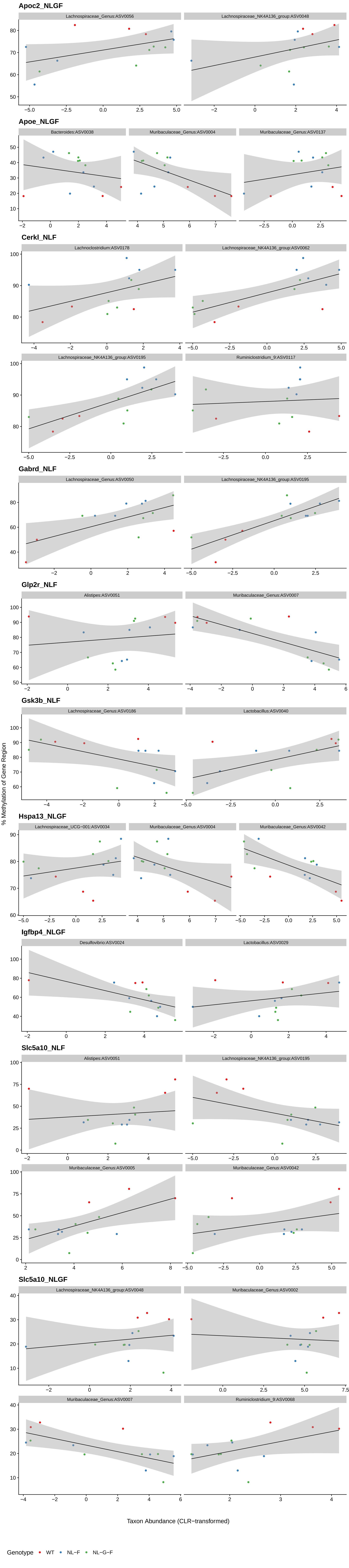

Apoc2_NLGF
Lachnospiraceae_Genus:ASV0056
Lachnospiraceae_NK4A136_group:ASV0048
Apoe_NLGF
Bacteroides:ASV0038
Muribaculaceae_Genus:ASV0004
Muribaculaceae_Genus:ASV0137
Cerkl_NLF
Lachnoclostridium:ASV0178
Lachnospiraceae_NK4A136_group:ASV0062
Lachnospiraceae_NK4A136_group:ASV0195
Ruminiclostridium_9:ASV0117
Gabrd_NLF
Lachnospiraceae_Genus:ASV0050
Lachnospiraceae_NK4A136_group:ASV0195
Glp2r_NLF
Alistipes:ASV0051
Muribaculaceae_Genus:ASV0007
Gsk3b_NLF
Lachnospiraceae_Genus:ASV0186
Lactobacillus:ASV0040
Hspa13_NLGF
Lachnospiraceae_UCG-001:ASV0034
Muribaculaceae_Genus:ASV0004
Muribaculaceae_Genus:ASV0042
Igfbp4_NLGF
Desulfovibrio:ASV0024
Lactobacillus:ASV0029
Slc5a10_NLF
Alistipes:ASV0051
Lachnospiraceae_NK4A136_group:ASV0195
Muribaculaceae_Genus:ASV0005
Muribaculaceae_Genus:ASV0042
Slc5a10_NLGF
Lachnospiraceae_NK4A136_group:ASV0048
Muribaculaceae_Genus:ASV0002
Muribaculaceae_Genus:ASV0007
Ruminiclostridium_9:ASV0068
% Methylation of Gene Region
Taxon Abundance (CLR-transformed)
Genotype
WT
NL-F
NL-G-F
