# Supplementary material for: Integrated analysis of behavioral, epigenetic, and gut microbiome analyses in AppNL-G-F, AppNL-F, and wild type mice
Source: Sci Rep. 2021 Feb 25;11:4678. doi: 10.1038/s41598-021-83851-4 (PMC7907263; doi:10.1038/s41598-021-83851-4)
Supplement: Supplementary file 8 — Supplementary Legends. [file 41598_2021_83851_MOESM8_ESM.pdf]

Integrated analysis of behavioral, epigenetic, and gut microbiome analyses in *App*$^{NL\text{-}G\text{-}F}$, *App*$^{NL\text{-}F}$, and wild type mice

Payel Kundu[1*], Eileen Ruth S. Torres[1*], Keaton Stagaman[2], Kristin Kasschau[2], Mariam Okhovat[3], Sarah Holden[1], Samantha Ward[3], Kimberly A. Nevonen[3], Brett A. Davis[3], Takahashi Saito[4], Takaomi C. Saido[5], Lucia Carbone[3, 6-8], Thomas J. Sharpton[2,9], Jacob Raber[1,10#]

Supplementary Table Legends

Table S1. Reduced Representation Bisulfite Sequencing of genome-wide patterns of DNA methylation in the hippocampus of the female *App*$^{NL\text{-}G\text{-}F}$, *App*$^{NL\text{-}F}$ and WT control mice show low levels of CHG and CHH methylation. H is A, T or C (CHG: 0.9% $\pm$ 0.26 and CHH: 1.1% $\pm$ 0.25; mean $\pm$ stdev), indicative of successful bisulfite conversion.

Table S2. Comparison of genome-wide DNA methylation levels in pair-wise comparisons of each of the *App*$^{NL\text{-}G\text{-}F}$ and *App*$^{NL\text{-}F}$ mice to age-matched wild-type controls. A total of 628 and 562 unique significant differentially methylated regions (DMRs; *q*-value < 0.05) were identified.

Table S3. Analysis of enrichment of gene ontology (GO) terms among DMR-containing genes. A significant enrichment of several GO terms related to AD was found among the *App*$^{NL\text{-}G\text{-}F}$ DMRs (for example, Regulation of long-term synaptic potentiation (GO:1900271) and Positive regulation of synaptic transmission (GO:0050806); *p*-value <0.05), but not among *App*$^{NL\text{-}F}$ DMR-containing genes. Some GO terms relevant to AD are indicated in orange.

Table S4. Significant Enrichment of DMR-containing genes in *App*$^{NL\text{-}G\text{-}F}$, but not *App*$^{NL\text{-}F}$, mice for genes associated with several AD-related phenotypes in the National Human Genome Research Institute catalog of published genome-wide association studies (NHGRI-GWAS).
